# Copper(II) *N*,*N*,*O*-Chelating Complexes as Potential Anticancer Agents

**DOI:** 10.1021/acs.inorgchem.0c02932

**Published:** 2021-02-17

**Authors:** Quim Peña, Giuseppe Sciortino, Jean-Didier Maréchal, Sylvain Bertaina, A. Jalila Simaan, Julia Lorenzo, Mercè Capdevila, Pau Bayón, Olga Iranzo, Òscar Palacios

**Affiliations:** † Departament de Química, Facultat de Ciències, Universitat Autònoma de Barcelona, 08193 Cerdanyola del Vallès, Barcelona, Spain; ‡ Aix Marseille Univ., CNRS, Centrale Marseille, iSm2, 13397 Marseille, France; § Institute of Chemical Research of Catalonia (ICIQ), Av. Països Catalans 16, 43007 Tarragona, Spain; ∥ Aix Marseille Univ., CNRS, IM2NP, 13397 Marseille, France; ⊥ Institut de Biotecnologia i Biomedicina, Departamento de Bioquímica i Biologia Molecular, Universitat Autònoma de Barcelona, 08193 Cerdanyola del Vallès, Barcelona, Spain

## Abstract

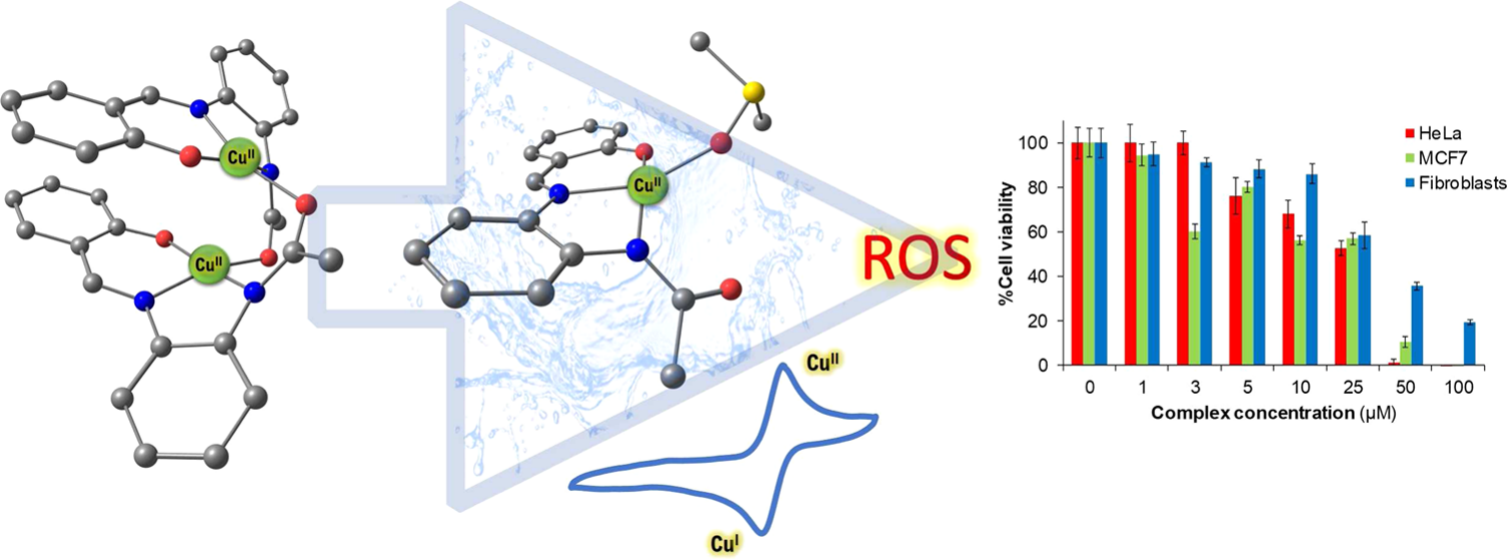

Three novel dinuclear Cu(II) complexes based on a *N*,*N*,*O*-chelating salphen-like ligand scaffold and bearing varying aromatic substituents (−H, −Cl, and −Br) have been synthesized and characterized. The experimental and computational data obtained suggest that all three complexes exist in the dimeric form in the solid state and adopt the same conformation. The mass spectrometry and electron paramagnetic resonance results indicate that the dimeric structure coexists with the monomeric form in solution upon solvent (dimethyl sulfoxide and water) coordination. The three synthesized Cu(II) complexes exhibit high potentiality as ROS generators, with the Cu(II)/Cu(I) redox potential inside the biological redox window, and thus being able to biologically undergo Cu(II)/Cu(I) redox cycling. The formation of ROS is one of the most promising reported cell death mechanisms for metal complexes to offer an inherent selectivity to cancer cells. In vitro cytotoxic studies in two different cancer cell lines (HeLa and MCF7) and in a normal fibroblast cell line show promising selective cytotoxicity for cancer cells (IC_50_ about 25 μM in HeLa cells, which is in the range of cisplatin and improved with respect to carboplatin), hence placing this *N*,*N*,*O*-chelating salphen-like metallic core as a promising scaffold to be explored in the design of future tailor-made Cu(II) cytotoxic compounds.

## Abstract 2

Three novel dinuclear Cu(II) complexes based on a *N*,*N*,*O*-chelating salphen-like ligand scaffold and bearing varying aromatic substituents (−H, −Cl, and −Br) have been synthesized and characterized. They three exhibit high potentiality as reactive oxygen species (ROS) generators, with the Cu(II)/Cu(I) redox potential inside the biological redox window. In vitro studies in two different cancer cell lines (HeLa and MCF7) and in a normal fibroblast cell line show promising selective cytotoxicity for cancer cells.

## Introduction

Metals and their inorganic complexes show an enormous versatility in front of strictly organic compounds for the development of therapeutic agents. The possibility of having several oxidation states, different coordination numbers, and diverse geometries gives rise to a broader spectrum of tuneable properties.^1^ Among them, Cu complexes have become promising alternatives for cancer treatment during the two last decades.^2−5^ Copper is a physiological metal, being widely present in many biomolecules and playing a remarkable role in a diversity of biochemical processes because of its interesting Cu(II)/Cu(I) redox pair.^6^ In fact, one of the main potentialities of Cu as an antiproliferative agent lies in its capability to form reactive oxygen species (ROS) inside the cells. The generation of these entities (H_2_O_2_, O_2_^•–^, HO^•^, etc.) is not only reported to damage DNA but also to offer a putative discrimination between healthy and cancer cells.^7,8^

The lack of selectivity in cancer therapy has always been a downside in this field, giving rise to severe side effects.^9^ Tumors contain a more reducing environment with respect to healthy tissues. This is based on what is known as “the Warburg effect” and consequence of the fact that cancer cells do primarily generate energy by an atypical aerobic glycolysis pathway.^10,11^ This abnormal metabolic process (instead of the usual oxidative phosphorylation) induces an imbalanced redox homeostasis inside cancer cells, leading to an enhanced intracellular ROS production.^12^ Consequently, the interference with cellular redox homeostasis arises as an attractive and promising target for chemotherapy. Cancer cells exhibit abnormal levels of ROS, and they show higher vulnerability to ROS level changes than healthy cells do; therefore, the alteration of those levels may be a unique opportunity to selectively target cancer cells.^7,8^ There is, hence, high potential for the development of bioreducible metal complexes.

Up to date, several Cu(II) complexes have been reported to be redox-active,^5,13−15^ and indeed, some structure–activity relationships have been reported between the redox behavior of *N*-donor aromatic Cu(II) complexes and their ROS-mediated cytotoxicity.^16−18^ In particular, Schiff-based Cu(II) complexes have attracted attention from researchers in this field and have been reported to show interesting cytotoxicity toward cancer cells and DNA cleavage.^15,19−21^ Not many Cu(II) complexes with *N*,*N*,*O*-chelating Schiff base ligands have been evaluated in cancer cells,^22^ in contrast to Cu(II) *N*,*N*,*S*-chelated thiosemicarbazone and bis(thiosemicarbazone) complexes.^23−27^

Here, we describe the synthesis, characterization, and evaluation of the biological activity of three novel Cu(II) complexes bearing *N*,*N*,*O*-chelating salphen-like ligands as potential antitumoral agents. The aim is to obtain biologically accessible Cu(II)/Cu(I) redox cycling systems, which would be able to generate high ROS levels in cells. This should lead to enhanced toxicity toward cancer cells with respect to healthy ones. The impact of halogenated substituents has also been evaluated and is discussed here. Speciation and the putative active species in solution are discussed hereby from a theoretical approach, and the mechanism of action is thoroughly evaluated and related to the redox behavior of the Cu(II) complexes.

## Results and Discussion

This work is based on the design of a basic scaffold ((*E*)-*N*-(2-(2-hydroxybenzylideneamino)phenyl)acetamide, **H**_**2**_**L1**), which specifically intends to chelate Cu(II) in a tridentate fashion having a fourth labile in-plane coordination position, with the idea of biologically attaining a fast Cu(II)/Cu(I) redox cycle.

### Synthesis and Characterization of the Ligands and Their Corresponding Copper(II) Complexes

**Scheme 1:**
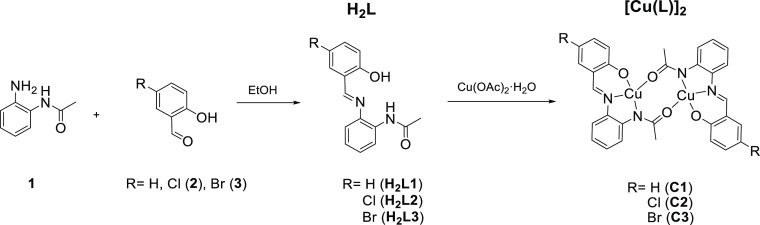
General Synthesis of the *N*,*N*,*O*-Chelating Salphen-like Ligands **H**_**2**_**L1** (R = H), **H**_**2**_**L2** (R = Cl), and **H**_**2**_**L3** (R = Br) and of Their Corresponding Cu(II) Complexes **C1**–**C3**

The three *N*,*N*,*O*-chelating salphen-like ligands (**H**_**2**_**L1**, **H**_**2**_**L2**, and **H**_**2**_**L3**, Scheme 1) were synthesized based on a condensation reaction between the mono-protected benzene-1,2-diamine precursor (**1**) and the corresponding salicylaldehyde precursor. Pure ligands were obtained by column chromatography purification. Characterization data are reported in the experimental section and Supporting Information (Figures S1–S3).

Mono-protected diamine (**1**) was obtained following the procedures reported in the literature.^28^ For **H**_**2**_**L1**, commercially available 2-hydroxybenzaldehyde was used as the starting material. To obtain **H**_**2**_**L2** and **H**_**2**_**L3**, halogen derivatization was carried out on the *para*-hydroxy position of the starting material 2-hydroxybenzaldehyde following the reported procedures. Chlorination was carried out under mild conditions using *N*-chlorosuccinimide (NCS) and an acid catalyst.^29^ This reaction provided lower yields than those found with common chlorinating agents (Cl_2_ or sulfuryl chloride), but a cleaner reaction.^30^ The final 4-chloro-2-hydroxybenzaldehyde (**2**) precursor was purified through column chromatography. Alternatively, bromination of the starting material was carried out using standard procedures with Br_2_, to obtain compound **3**.

Complexation of pure **H**_**2**_**L1**–**H**_**2**_**L3** was carried out using Cu(OAc)_2_ as the metal precursor salt (Scheme 1). The use of the Cu(II) acetate salt allowed deprotonating −*OH* and −*NH* at once. Complexes (**C1**–**C3**, Scheme 1) were isolated as brownish powders by precipitation from the reaction media. In all the cases, the solubility of the complexes was very poor in the common organic solvents, especially for **C2** and **C3**, even if this can be improved by the use of coordinating solvents such as dimethyl sulfoxide (DMSO) and dimethylformamide.

The data recorded for the complexes (Experimental Section, Figure S4 and Table S1) suggest a 1:1 ligand to metal stoichiometry in the solid state (elemental analysis, see Experimental Section), with both −OH and −NH groups deprotonated and in the absence of any additional ligand or counterion. The IR bands of **H**_**2**_**L** (Figures S1C–S3C) assigned to the stretching of both *O*–*H* (phenol) and *N*–*H* (amide) bonds at 3500–3300 cm^–1^ as well as those related to the bending mode of the *N*–*H* bond (about 1660 cm^–1^, scissor bending) and of the *O*–*H* (1500 cm^–1^) bond have disappeared in the corresponding Cu(II) complexes (Figure S4A). This confirms the deprotonation of both the amide (−N*H*) and the phenol (−O*H*) groups upon metalation. No peaks for additional counterions or ligands have been observed. The Cu(II) coordination sphere in the solid state is composed of the *N*,*N*,*O*-chelating salphen-like ligand and a fourth oxygen from the carbonyl of the amide group (C=O) of the second entity of the dimer.

**Figure 1:**
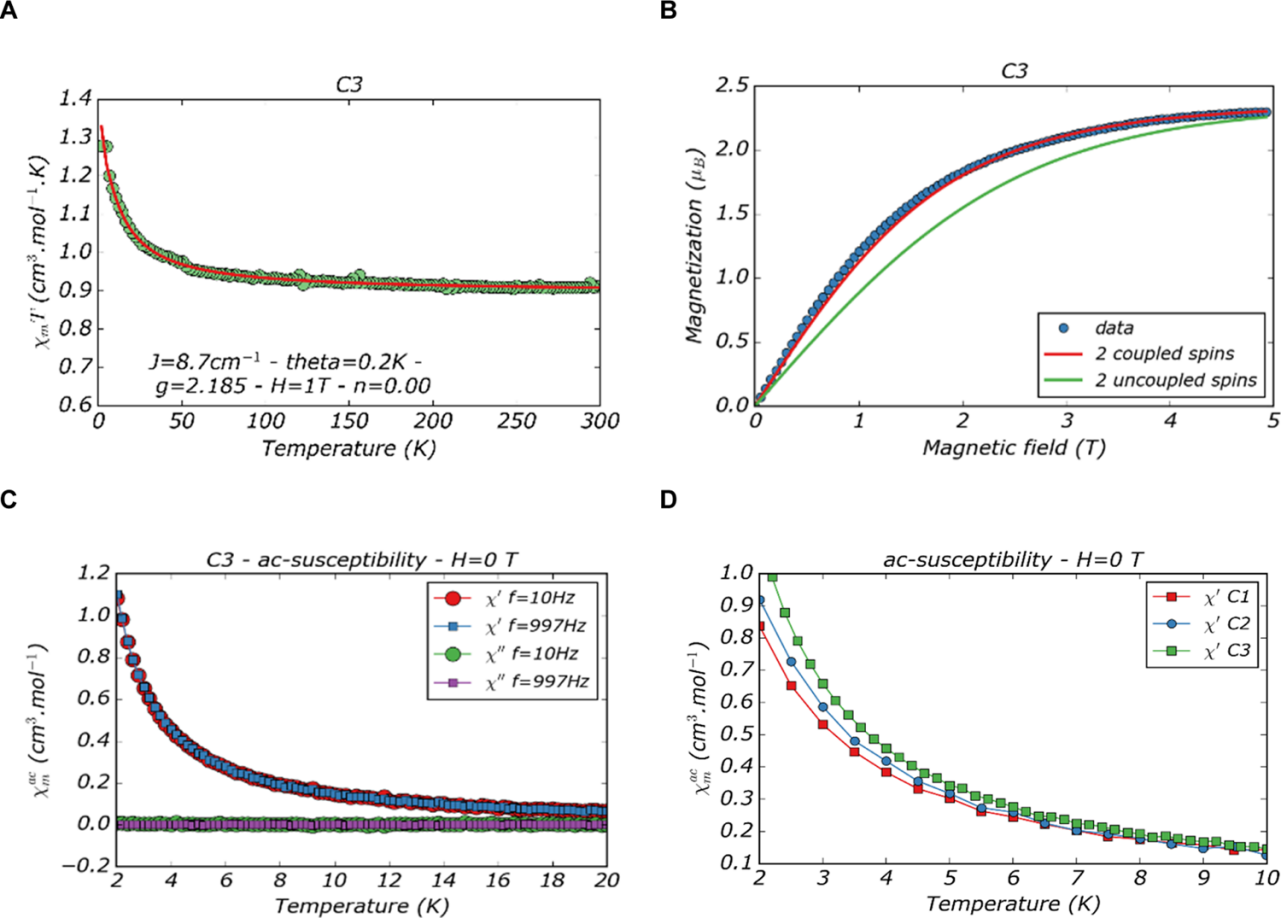
SQUID data obtained for complex **C3**: (A) susceptibility measured at 1 T. Red curve is the best fit for a dimeric complex, (B) magnetization measured at 2 K showing a fitting with the theoretical model for 2 coupled spins, and (C) AC—magnetic susceptibility. (D) Comparison of the magnetic susceptibility of **C1**–**C3** complexes.

The magnetic properties of the complexes **C1**–**C3** were studied using a conventional SQUID magnetometer. The results for the three complexes are very similar. The results for **C3** are presented in Figure 1, while those for **C1** and **C2** are given in the Supporting Information (Figure S5). Figure 1A shows the isofield (*H* = 1 T) χ*T* as a function of temperature (*T*). The red curve is the best fit using the Bleaney–Bowers equation of a coupled *S* = 1/2 dimer.^31^ The fit shows a ferromagnetic coupling for the three complexes, with small exchange coupling constant (*J*) values ranging from 3.5 to 8.7 cm^–1^ and a *g*-factor of 2.15 to 2.2 coherent with the presence of Cu(II) (Table 1) and the absence of monomers. The increase in the *J* values (Table 1) with the increase of the size of the *R* substituent (Scheme 1) suggests that the functionalization influences the Cu(II)–Cu(II) distance in the dinuclear structure. Additionally, the very similar *g* values obtained points to an analogous conformation for all three complexes. Isothermal magnetization (*T* = 2 K) confirms the presence of two coupled spins (Figures 1B and S5). Finally, AC-susceptibility measurements (Figures 1C and S5) indicate the absence of long-range order that would be due to the presence of polymers.

**Table 1:** Experimental *J*_Cu–Cu_ (cm^–1^) and *g*-Factors Obtained from SQUID Measurements in the Solid State for Complexes **C1**–**C3**

	*J*_Cu–Cu_ (cm^–1^)	*g*
**C1**	3.5	2.19
**C2**	5.7	2.15
**C3**	8.7	2.19

The integrity of the species was evaluated in the DMSO solution. The dimeric structure of **C1**–**C3** has been confirmed by the observation of the corresponding peaks in HR-ESI-MS (*m*/*z* 631.0456, 700.9661, and 786.8678, respectively, Figure S4A). These data indicate that the nuclearity is at least partially maintained in solution. At the same time, peaks attributed to the corresponding mononuclear species were also found (Figure S4C), suggesting a solvent-dependent process involving partial breakage of the dinuclear species and coordination of the DMSO solvent in the fourth binding site of the metal coordination sphere. Electron paramagnetic resonance (EPR) shows the presence of a single EPR-active Cu(II) species in solution for all the three complexes (Figure S4B and Table S1). The observed EPR signals for **C1**–**C3** in the DMSO solution are typical for Cu(II) monomeric species in square–pyramidal-derived geometries with the single electron in d_*x*^2^–*y*^2^_ orbitals (*g*_∥_ > *g*_⊥_ > *g*_e_). From the analysis of the EPR parameters derived from simulations (*A* and *g*-tensors), it appears that the three complexes mainly exist in a nonsignificantly distorted square-planar or square-pyramidal geometries with the N_2_O_2_ coordination in the equatorial plane, as expected based on the *N*,*N*,*O*-chelating ligands and on solvent coordination. In order to provide further insights into the monomer/dimer coexistence of the complexes in DMSO, quantification of the Cu(II) mononuclear species through double integration of the EPR spectra of **C1**, as the model compound, was carried out at different time points (Table S2). EPR spin quantification data demonstrate that the dissolution of **C1** in DMSO gives rise to 30% of the mononuclear Cu(II) signal, thereby pointing to the presence of EPR-silent magnetically coupled dinuclear species. The Cu(II) signal evolves over time reaching about 50% of the mononuclear species after 24 h. This confirms the dimeric cleavage process in a solvent and time-dependent manner. In addition, increasing the ionic strength by the addition of salts seems to slightly contribute to the cleavage of the dimeric form (Table S2) reaching up to 60% after several days. The overall data are thus in concordance with those of ESI-MS analysis (Figure S4C), and suggest the coexistence of both dimer and monomer species in solution.

### DFT Studies for the Evaluation of the Active Species in Solution

**Figure 2:**
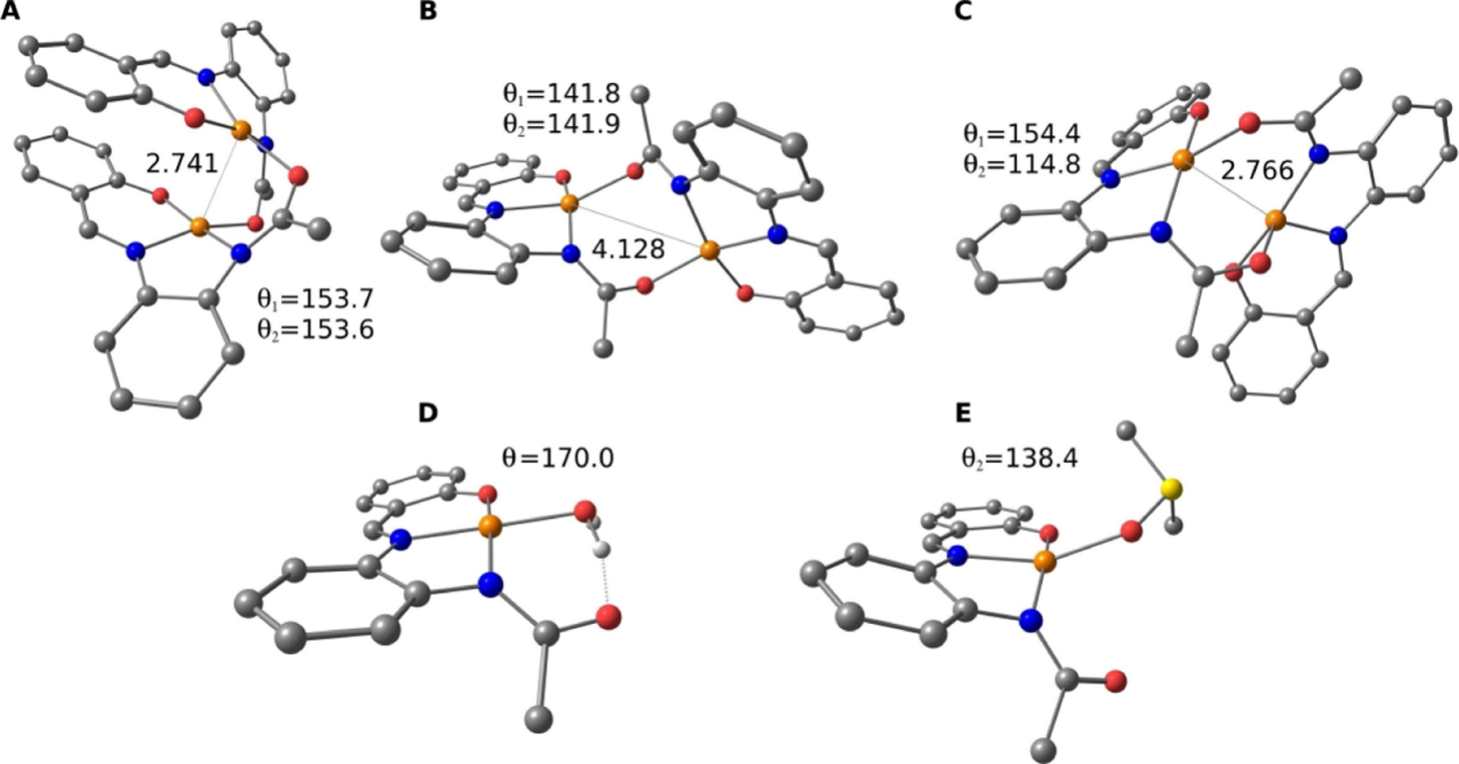
(A–C) Optimized geometry of the three main conformations of the dimeric complex [Cu^II^(**L1**)]_2_, and the monomeric species (D) [Cu^II^(**L1**)(H_2_O)] and (**E**) [Cu^II^(**L1**)(DMSO)]. Cu–Cu distances are reported in angstrom. The deviation from square-planar toward tetrahedral geometry is also reported in degrees as the dihedral angle (θ) between the fourth equatorial donor atom and the donors of the tridentate chelating **L1** ligand (D–N_cis_–O–N_trans_).

Density functional theory (DFT) computational studies of the parent ligand **H**_**2**_**L1** and its corresponding Cu(II) complex **C1** have been carried out to model and rationalize the speciation in solution of **C1**–**C3** complexes. Two solvents were chosen to be computed: DMSO to compare with the beforehand obtained experimental values and water because of its biological relevance. Dimeric and monomeric Cu(II) complexes formed by the ligand **H**_**2**_**L1** (Scheme 1) were examined and their proposed structures were simulated: [Cu^II^(**L1**)]_2_, [Cu^II^(**L1**)(H_2_O)], and [Cu^II^(**L1**)(DMSO)] (Figure 2). Concerning the dimeric species, among the seven different conformations considered with relative orientation of **L1** ligands and the coordination position (axial or equatorial) of their donors, only three have been characterized as minima in the potential energy surface (Figure 2A–C).

In all the cases, the Cu(II) centers present a square-planar arrangement with different grades of distortion, ranging from almost pure square-planar geometries for [Cu^II^(**L1**)(H_2_O)] (170.0°, Figure 2D) to highly distorted for [Cu^II^(**L1**)]_2_ (114.8° in conformation C, Figure 2C).

The Gibbs energy calculations for the dissociation reactions of the dimeric forms to monomeric species (Table 2) suggest a different behavior depending on the solvent. In water, the dissociation reaction appears to be favored with Δ*G*_aq_ from −1.8 up to −12.6 kcal·mol^–1^, while in DMSO, data are consistent with the coexistence of the dimeric and monomeric forms with Δ*G*_aq_ from 4.4 to −1.0 kcal·mol^–1^.

**Table 2:** Δ*G* Values for the Dissociation of the Dimeric Species [Cu^II^(**L1**)]_2_ to the Monomeric Complex [Cu^II^(**L1**)(solv)] in Different Solvents^a^

[Cu^II^(L1)]_2_	solv	Δ*E*_solv_	Δ*G*_solv_
conformation A	H_2_O	–1.8	–1.8
	DMSO	9.2	4.4
conformation B	H_2_O	–9.5	–9.2
	DMSO	6.9	3.7
conformation C	H_2_O	–12.6	–11.4
	DMSO	2.8	–1.0

a Values reported in kcal mol^–1^. The corrections of *RT* ln *V* (1.89 kcal/mol) and *RT* ln([solv]/*n*) were applied. Values computed in the SMD continuum model for H_2_O or DMSO.

To corroborate the structures obtained and to discriminate between the three dimeric forms, the *J* values were computed in each case to determine the ferro or antiferromagnetic nature of the interaction between the two unpaired electrons on the Cu(II) centers (Table 3), and compared with those obtained experimentally (Table 1). Computed values show that only conformation B (Figure 2B) has an antiferromagnetic coupling (*J* = −43.2 cm^–1^). In contrast, for conformations A and C (Figure 2A,C), the predicted interaction is ferromagnetic (12.0 and 22.3 cm^–1^, respectively). The reason behind the different magnetic behaviors can be found from the Cu–Cu distances between the two radical spins, that is, ∼2.7 Å in conformations A and C allowing a ferromagnetic coupling, versus 4.1 Å in conformation B, in which the cores are well separated (Figure 2).^32^ According to the experimental ferromagnetic exchange couplings for the three complexes (*J*_Cu–Cu_, Table 1), the most probable structure of **C1**–**C3** would fit with conformation A, which shows the lowest Gibbs energy and *J*_Cu–Cu_ values (Table 3).

**Table 3:** Simulated *J*_Cu–Cu_ (cm^–1^) and Δ*G* Values for the Different Conformations of the Dimeric [Cu^II^(**L1**)]_2_ Species^a^

[Cu^II^(L1)]_2_	*J*_Cu–Cu_ (cm^–1^)	coupling	Cu–Cu distance (Å)	Δ*G*_DMSO_/Δ*G*_aq_ (kcal·mol^–1^)^b^
conf. A	12.0	ferromagnetic	2.741	0.0/0.0
conf. B	–43.2	antiferromagnetic	4.128	0.7/7.4
conf. C	22.3	ferromagnetic	2.766	5.3/9.7

a *J* has been determined with a reported method.^33,34^

b DFT level using B3LYP-D3 combined with the basis-set def2-TZVP for the main group elements and the quadruple-ζ def2-QZVP basis set for Cu.

The EPR parameter simulations for the monomeric species in DMSO, [Cu^II^(**L1**)(DMSO)], are in the range of the experimental results (Table 4). The relative deviation of the calculated *g*_*z*_ and *A*_*z*_ values from the experimental ones is −16.0% for *A*_*z*_ and −1.9% for *g*_*z*_. The larger deviation of *A*_*z*_ for [Cu^II^(**L1**)(DMSO)] must be related to the significant distortion of the equatorial plane of the Cu(II) ion because of the coordination of DMSO (θ = 138.4°), and these differences are common between the computed and experimental parameters, especially on the *A* tensor.^35^

**Table 4:** EPR Parameters Computed (Calc) for the Monomeric [Cu^II^(**L1**)(DMSO)] Species in the DMSO Medium, and Comparison with the Experimental (Exp) Values [Relative Deviation (RD) Related to the Experimental Value], Extracted from Figure S4B and Table S1

species	*A*_*z*_^calc,^^a^	*A*_*z*_^exp,^^a^	*A*_*z*_^RD^	*g*_*z*_^calc^	*g*_*z*_^exp^	*g*_*z*_^RD^
[Cu^II^(**L1**)(DMSO)]	154.0	183.4^b^	16.0%	2.201	2.244^b^	1.9%

a Values in 10^–4^ cm^–1^.

b Values recorded in DMSO.

The UV–vis vertical excitation has also been computed for both dimeric [Cu^II^(**L1**)]_2_ (conformation A) and monomeric [Cu^II^(**L1**)(H_2_O)] and [Cu^II^(**L1**)(DMSO)] species and compared with experimental values (Figures S6 and S7 and Table S3). Computed MLCT transition bands are in the range of the experimental ones for the monomeric [Cu^II^(**L1**)(solv)] species (Figure S6 and Table S3), while computed Cu(II) d–d transitions could indeed fit with both forms (dimer and monomer, Figure S7 and Table S3). The overall results are in concordance with the EPR values compared previously. Even if the presence of the dimeric form has been widely demonstrated, all data point to a significant role of the monomeric Cu(II) form in the final activity of **C1** in solution.

### Evaluation of the Potentiality of the Complexes as ROS Generators

**Figure 3:**
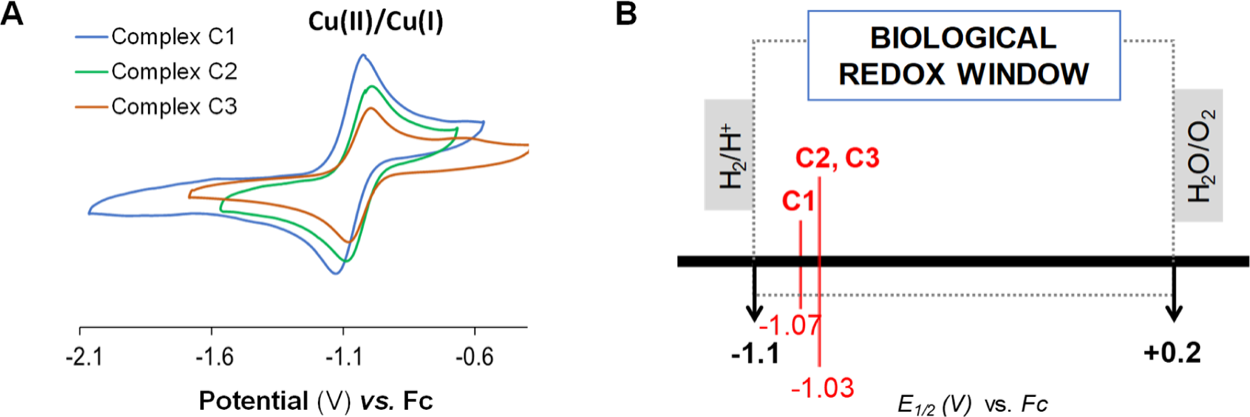
(A) Cyclic voltammograms vs Fc^+^/Fc (Fc) of **C1**–**C3** in DMSO with 0.1 M TBAP at a scan rate of 100 mV/s. (B) Representation of *E*_1/2_ values obtained for **C1**–**C3** placed in the biological redox window at pH 7.^36^

The redox properties of the Cu(II) complexes were evaluated by cyclic voltammetry (CV) experiments. CV was carried out with both the ligands (**H**_**2**_**L1**–**H**_**2**_**L3**) and the complexes (**C1**–**C3**) in DMSO. Taking into account that the biological redox window approximately ranges from −1.1 to 0.2 V versus Fc^+^/Fc (values arising from the oxidation and reduction of water at pH 7,^36^ respectively), we specifically analyzed in detail this region (Figure 3). **H**_**2**_**L1**–**H**_**2**_**L3** do not show any kind of redox activity in this specific range. On the contrary, all the Cu(II) complexes are redox active and the signals observed on the cyclic voltammograms of **C1**–**C3** have been ascribed to the Cu(II) ⇄ Cu(I) redox process (Figure 3).

The redox potentials (Table S4) were assigned to the redox couple Cu(II)/Cu(I) based on bulk electrolysis and EPR experiments (data not shown). The difference between cathodic and anodic peaks in **C1**–**C3** cyclic voltammograms is higher than the theoretical 0.060 V for fully reversible redox processes (Δ*E*_p_ ranging from 0.11 to 0.16 V), but in the range of the Δ*E*_p_ (0.10–0.12 V) obtained for the ferrocene (Fc^+^/Fc) reference compound under the same experimental conditions. Successive scans were performed, and the lack of signal change upon the successive collected scans indicates that no disproportion occurred after cycling between Cu(II) and Cu(I) in none of the three Cu(II) complexes. The *I*_pa_/*I*_pc_ ratio close to 1 and the calculated Δ*E*_p_ values (Table S4) suggest a quasireversible one-electron process. The linear dependence of the peak currents *I*_pc_ and *I*_pa_ versus the square root of the scan rate (ν^1/2^) is indicative of a diffusion-controlled process (Figure S8).^37^

The determined Cu(II)/Cu(I) redox potentials (*E*_1/2_ = −1.07 V for **C1** and −1.03 for **C2**–**C3** vs Fc^+^/Fc, Table S4) are within the biological range of −1.1 to 0.2 V versus Fc^+^/Fc (Figure 3B). The presence of electrowithdrawing groups in **C2** and **C3** slightly favors the Cu(II) reduction to Cu(I) (*E*_red_ = −1.09 and −1.08 V, respectively) compared to **C1** (*E*_red_ = −1.15 V) (Table S4). Both the chloro- and bromo-derivatives have the reduction potential 60 and 70 mV higher than **C1**. Despite the fact that halogen groups make Cu(II) more prone to be reduced to Cu(I), the final *E*_1/2_ values for the three complexes are similar (Figure 3B and Table S4).

In order to characterize the center of the redox process, the Gibbs energy of the product of the monomeric **C1** reduction process was calculated at the DFT theory level for two different spin multiplicities: *S* = 1, corresponding to [Cu^I^(**L1**)(DMSO)]^−^, and *S* = 3, accounting for the **L1** reduction forming [Cu^II^(**L1**^•–^)(DMSO)]^−^ (Figure S9).^18^ The obtained Gibbs free energy value of the reduction on the ligand is 32.7 kcal·mol^–1^ higher than that on the metal center (Table S5). This difference highlights that the ligand participation in the redox process is negligible and the oxidation state of Cu in the two minima can be described as +II and +I.

**Figure 4:**
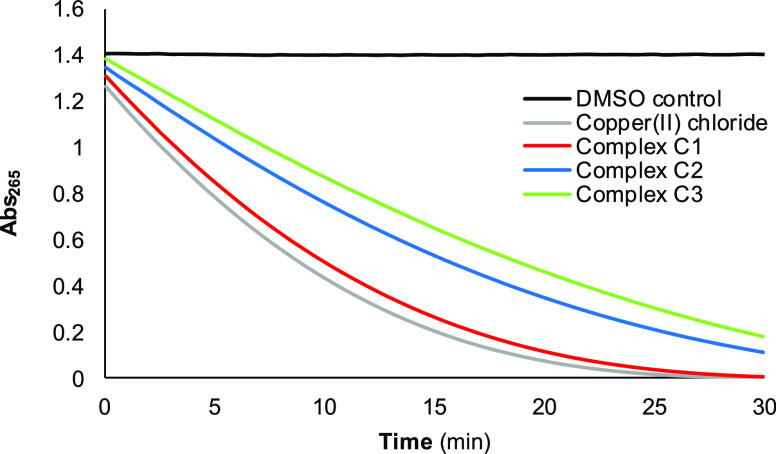
Consumption of ascorbate (100 μM) mediated by CuCl_2_ and complexes **C1**, **C2**, and **C3** in NaCl and Tris–HCl buffer at pH 7.2 (5% DMSO). The four Cu(II) compounds were at a concentration of 2 μM.

The CV results suggest that the **C1**–**C3** complexes can be thermodynamically reduced by biological redox buffers, and perform a quasireversible redox process. Therefore, they seem to be capable of undergoing Cu(II)/Cu(I) redox cycling under biological conditions. In order to confirm their capability to biologically undergo Cu(II)/Cu(I) redox cycling, ascorbate consumption at pH 7.2 was monitored by UV–vis (Figure 4). Cu(II), in the presence of ascorbate and under aerobic conditions, catalyzes the generation of ROS.^38^ Measuring the consumption of ascorbate at its maximum absorbance (265 nm) in the presence of the Cu(II) complexes provides an idea of their capability to generate ROS inside cells. In the absence of any Cu catalyst (DMSO control), no decrease in the absorbance at 265 nm can be observed (Figure 4), thus indicating that ascorbate (100 μM) is stable and the medium does not consume it. In contrast, the presence of a catalytic amount of free Cu(II) ions (2 μM of CuCl_2_ addition) clearly shows a rapid decrease in the absorbance, and ascorbate has been almost totally consumed after just 20 min. Complex **C1** (2 μM concentration added) is able to consume it at similar rates than free copper(II) ions do, while **C2** and **C3** (at the same concentration as **C1**) exhibit a slower consumption rate than that of **C1**. One possible explanation for the different consumption rates could be related to solubility issues because **C2** and **C3** are less soluble in aqueous media than **C1**. Consequently, and based on the overall ascorbate consumption data, it is expected that **C1**–**C3** can exert some kind of redox-mediated cytotoxicity through the generation of ROS inside cells.

### Cytotoxicity Assays in Cancer and Normal Cell Lines

The in vitro antiproliferative activity of the complexes **C1**–**C3** and their corresponding free ligands were determined on somatic HeLa and MCF7 cancer cell lines (Table 5 and Figure S10).

**Table 5:** IC_50_ (μM) Values at 72 h of Complexes **C1**, **C2**, **C3** and Their Corresponding Ligands in HeLa, MCF7, and NIH 3T3 Cultures, Using CuCl_2_·2H_2_O as the Reference Compound^a^

compound	HeLa	MCF7	NIH 3T3
**C1**	26 ± 4	30 ± 6	≥100
**C2**	25 ± 2	b	b
**C3**	23 ± 10	29 ± 5	b
**H**_**2**_**L1**	≥200	≥150	≥200
**H**_**2**_**L2**	≥50	b	b
**H**_**2**_**L3**	≥50	≥50	b
CuCl_2_·2H_2_O^18,39^	≥200	≥200	≥200

a The results shown are representative of at least three independent experiments (*N* = 3).

b Experiments were not carried out because of poor solubility in the cell culture medium. In the case of the nonassayed complexes, their corresponding ligands were not assayed either.

The IC_50_ values obtained in HeLa cancer cells (Table 5 and Figure S10) for the ligands show that while ligand **H**_**2**_**L1** presents poor or negligible toxicity, **H**_**2**_**L2** and **H**_**2**_**L3** display significant cytotoxicity. This difference might be attributed to the presence of the halogen substituents.^40,41^ Complexes **C1**, **C2**, and **C3** exhibit remarkable and dose-dependent cytotoxicity in both HeLa and MCF7 cells (IC_50_ about 25 μM, Table 5) when compared to CuCl_2_ and to the two commercially available Pt-drugs cisplatin (IC_50,72h_ of 15 μM in HeLa^39^) and carboplatin (IC_50,72h_ of 39 μM in HeLa^42^). Both **C2** and **C3** are bearing toxic ligands (**H**_**2**_**L2** and **H**_**2**_**L3**), whereas **C1** does show significant antiproliferative activity, yet bearing a nontoxic ligand (**H**_**2**_**L1**). The toxicity of the latter can then only be attributed to a conjoint contribution between the ligand **H**_**2**_**L1** and the Cu(II) ion, that is, to the entire complex; and not solely to the simple addition of the Cu(II) ion plus the ligand toxicities. In the case of **C1**, this feature may imply an advantage in terms of drug metabolism because none of the frameworks that constitute the complex (**H**_**2**_**L1** and Cu(II) ion) do separately exhibit cytotoxicity.

**Figure 5:**
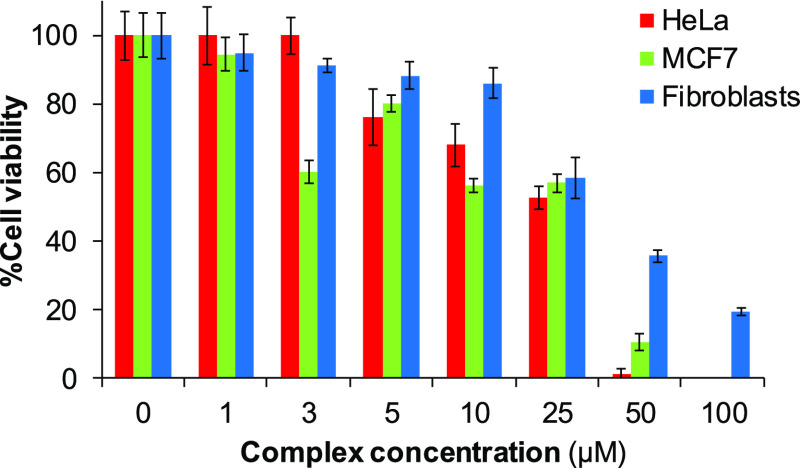
Comparison of the dose–response cell viability diagrams of **C1** in HeLa, MCF7, and NIH 3T3 (fibroblasts) cell lines (0–100 μM) at 72 h. The obtained values average at least three independent experiments.

Because of the low solubility in the biological culture medium exhibited by **C2** and, at lesser extent, **C3**, and considering the similar IC_50_ values in both cancer cell lines with that of **C1**, the latter was chosen as the model scaffold to evaluate the cytotoxicity toward normal embryotic fibroblasts (NIH 3T3), selected as nontumoral cell lines. As observed in the dose–response cell viability diagram (Figure 5), complex **C1** exhibits lower toxicity toward normal fibroblasts with respect to both HeLa and MCF7 cancer cells. This is interesting in terms of selective chemotherapy because it might provide less side effects.

### Evaluation of the Interactions of the Complexes toward DNA

In order to evaluate the effect and interaction of the complexes **C1**–**C3** with DNA, traditionally considered as one of the main targets of chemotherapy, several experiments have been carried out, namely gel electrophoresis, UV–vis, and/or circular dichroism (CD).

**Figure 6:**
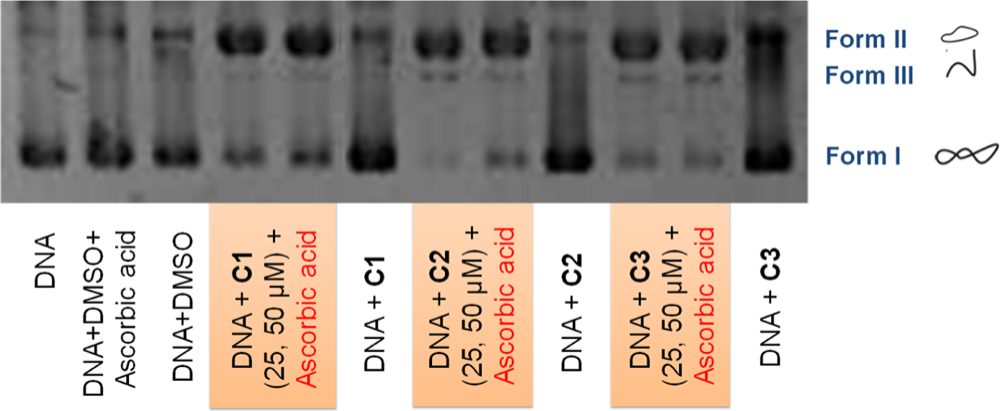
Agarose gel electrophoresis of a BlueScript Supercoiled DNA (ScdsDNA) treated with complexes **C1**, **C2**, and **C3**. Incubation time of 24 h at 37 °C. Some samples were incubated for an additional 1.5 h in the presence of ascorbic acid.

First of all, the cleaving properties of complexes **C1**, **C2**, and **C3** were investigated by gel electrophoresis because many Cu(II) complexes have been reported to induce cell death through DNA cleavage.^5,19,43,44^ The conversion of supercoiled circular plasmid DNA to open DNA forms was followed (Figure 6) and the obtained results indicate that the three complexes are only able to partially cleave supercoiled plasmid DNA (ScdsDNA), leading into a minor band corresponding to its open circular form (ocDNA, form II). This confirms that they do not possess prominent cleaving capacity by themselves. In contrast, the presence of a reductant species, such as ascorbic acid (a biological reductant), enhances their cleaving capacity (Figure 6, colored lines), and they are then able to practically transform all the ScdsDNA into ocDNA and, to a lesser extent, into its linear form (form III). As already mentioned, the generation of Cu(I) stimulates the potential formation of ROS, which have DNA-cleaving abilities.^3^ In our particular case, the results clearly point to a redox-dependent mechanism, triggered by the presence of ascorbic acid, which promotes the Cu(I) generation, the potential formation of ROS, and the concomitant DNA damage.

**Figure 7:**
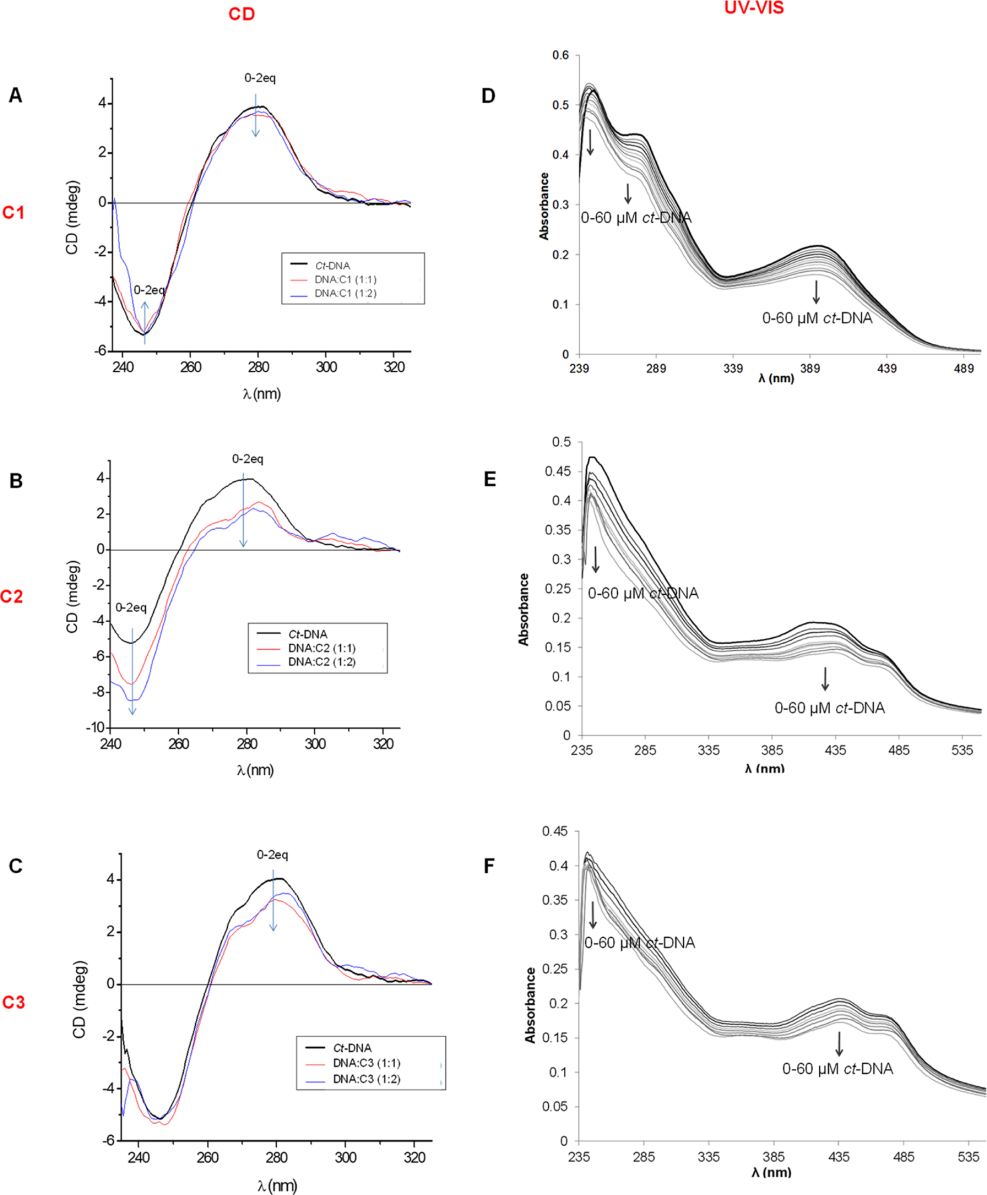
DNA-binding studies. On the left, the results of CD studies for **C1** (A), **C2** (B), and **C3** (C) at 50 μM of ct-DNA and at 1:1 and 1:2 (DNA/complex) ratios in NaCl/Tris–HCl at pH 7.2. Samples were previously incubated overnight at 37 °C. On the right, the results of UV–vis studies for complexes **C1** (D), **C2** (E), and **C3** (F) at 30 μM of each complex upon ct-DNA titration from 0 to 60 μM in NaCl/Tris–HCl at pH 7.2. Each spectrum was recorded after 15 min of stabilization time. The arrows indicate change upon increasing concentrations of ct-DNA.

Next, the binding of **C1**–**C3** with calf thymus DNA (ct-DNA) was studied. Covalent interactions with DNA are highly important in the case of cisplatin and Pt compounds,^45,46^ whose mechanism of action is usually conceived through the formation of Pt-DNA adducts. In the case of Cu(II) complexes, covalent adducts with DNA are less common and normally they do not show this kind of binding.^18^ CD and UV–vis spectroscopies have been used to enlighten the putative DNA-complex binding modes of **C1**, **C2**, and **C3** (Figure 7).

CD spectroscopy allows assessing the possible structural alterations of the characteristic bands of ct-DNA (a positive band around 280 nm and a negative band around 245 nm)^47^ upon complex interaction. ct-DNA (50 μM) was incubated with **C1**, **C2**, or **C3** (from 0 to 2 equivalents) overnight and analyzed by CD spectroscopy (Figure 7A–C). In all cases, only minor modifications of the initial CD signals were observed, pointing to slight structural changes in the helicity of the ct-DNA. This suggests some kind of noncovalent interaction, but without significant structural DNA modifications.

Three main classes of noncovalent binding have been proposed for metal complexes: intercalation, groove binding, and electrostatic interactions with the negatively charged phosphate backbone of DNA. In order to assess the nature of the complex–DNA interactions, UV–vis spectroscopy has been used to monitor the changes in the absorbance of **C1**–**C3** complexes upon increasing additions of ct-DNA to a solution of the corresponding metal complexes. Absorption spectra in the range of 225–550 nm were recorded at a constant complex concentration (30 μM) with increasing amounts of DNA. The results for complexes **C1**–**C3** (Figure 7D–F) clearly show a hypochromic effect upon ct-DNA addition, but no significant bathochromism is observed in any spectra. This points to an interaction with DNA via groove binding or electrostatic interactions rather than via intercalation.^48,49^ Compounds displaying high DNA-intercalating capabilities usually induce a bathochromic shift because of their π–π interactions with the aromatic bases of DNA, a phenomenon that has not been observed in this case.

Quantitative data, that is, the intrinsic binding constant *K*_b_, can be obtained from the recorded absorption spectra using the Benesi–Hildebrand equation (eq 1).^50^*A*_o_ is the absorbance of the complex in the absence of DNA, *A* is the absorbance at any given DNA concentration, and ε_G_ and ε_H–G_ are the extinction coefficients of the complex and the complex–DNA, respectively.
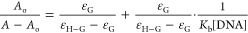
1

The plot of the relative variation of the absorbance (*A*_o_/(*A* – *A*_o_)) versus the inverse of the DNA concentration (1/[DNA]) (Figure S11) allows the determination of *K*_b_ (Table 6). The *K*_b_ values obtained for complexes **C1**, **C2**, and **C3** are in the order of 10^4^ M^–1^, indicating a moderate interaction and lower than the values around 10^6^ to 10^7^ known for classical and strong mtallointercalators (DAPI, HOECHST, etc.).^48,51,52^

**Table 6:** Intrinsic Binding Constants (*K*_b_) and Hypochromism for the Interaction of ct-DNA with Complexes **C1**, **C2**, and **C3**

Complex	*K*_b_ (M^–1^)^a^	log *K*_b_	% hypochromism (λ in nm)
**C1**	2.2 × 10^4^	4.34	25 (397)
**C2**	6.2 × 10^4^	4.79	27 (424)
**C3**	7.2 × 10^4^	4.86	28 (438)

a *K*_b_ is obtained from the ratio of the intercept to the slope, according to the Benesi–Hildebrand equation (eq 1),^48^ after the fitting of the UV–vis data (Figure 7D–F). The calculated *K*_b_ values arise from a DNA–drug interactions according to the Benesi–Hildebrand model (which gives approximated *K*_b_ values), and hence they should be compared in orders of magnitude, rather than with the exact numbers.

### In Vitro ROS Generation and Induction of Apoptosis

The results obtained from the CV studies (Figure 3), ascorbate consumption experiments (Figure 4), and DNA-cleaving activity (Figure 6) strongly indicate an oxidative dependent mechanism of action. In order to confirm the formation of intracellular ROS in HeLa cancer cells, the 2′,7′-dichlorofluorescin diacetate (DCFDA) assay was performed.^14,53^ DCFDA is a nonfluorescent and permeable dye that, after cleavage by intracellular esterases and subsequent oxidation by ROS, generates dichlorofluorescein (DCF), a fluorescent and nonpermeable compound.

**Figure 8:**
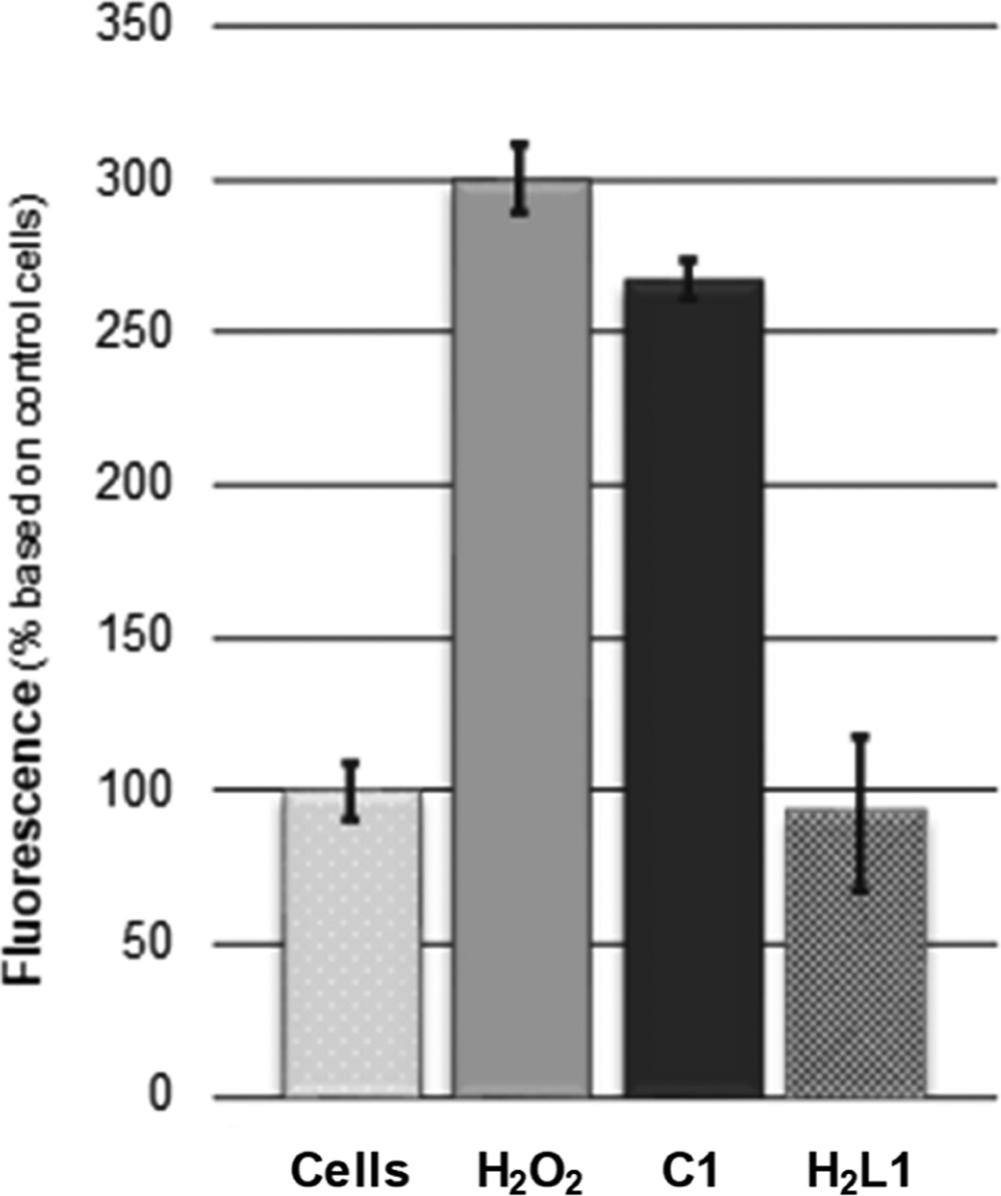
In vitro ROS production measured with the DCFDA assay in HeLa cancer cells for complex **C1** (25 μM), **H**_**2**_**L1** (50 μM), and H_2_O_2_ (100 μM) as positive control after treatment for 4 h.

The experiment was performed with **C1** as the main scaffold, and to serve as a proof-of-concept to understand the mechanism of action of the *N*,*N*,*O*-chelating metallic core. After 4 h treatment, strong DCF fluorescence, of up to 3-fold respect to control cells, was observed for **C1** (Figure 8), highlighting the ROS production capabilities of this Cu(II) complex. The ROS levels of **C1** are equivalent to those produced by the positive control H_2_O_2_. On the contrary, **H**_**2**_**L1** was not able to increase the ROS levels (Figure 8) with respect to the control group. This is in concordance with the Cu(II)/Cu(I) redox potential of **C1** (Figure 3) and with the results obtained for the toxicity of **H**_**2**_**L1** and **C1** (Table 5).

These results confirm the relationship inferred between the Cu(II)/Cu(I) redox potential of **C1**, its ROS production inside the cells, and the exerted biological activity. Furthermore, this ROS cell death pathway might explain the different toxicity profiles observed for **C1** in HeLa and MCF7 cancer cells with respect to normal cell lines (NIH 3T3) (Figure 5). Taking into account that cancer cells have higher radical levels than healthy ones, the production of ROS might appear as a differentiating feature. Accordingly, **C1** displays a lower toxicity profile in fibroblasts than in the two tested cancer cell lines (Figure 5).

Finally, the evaluation of the mechanism of cell death in HeLa cancer cells by **C1** was carried out by using the standard propidium iodide (PI)/Annexin V-Alexa Fluor 488 assay (Table S6). The induction of ROS has been related to the mechanism of apoptosis,^54^ and many research efforts have been devoted to the synthesis of potential anticancer agents that induce an apoptotic cell death pathway.^55,56^ The results indicate that **C1** is able to partially trigger apoptosis in HeLa cancer cells, where at least about 12% of cells are in the early apoptotic stage (Table S6). This value is in the range of cisplatin, which is well known to induce an apoptotic pathway.^57,58^ The rest of death cells (24%) have high fluorescence values of PI, indicating that the membrane is not intact. This might point to a necrosis, with loss of membrane integrity, or to an apoptotic necrosis (late apoptosis). This last mechanism involves an early apoptosis, which ends up (with time and in the absence of phagocytosis) in the membrane lysis of the already formed apoptotic bodies and in the organelle breakdown.

## Conclusions

In summary, three novel Cu(II) complexes have been synthesized with different *N*,*N*,*O*-chelating salphen-like ligands bearing varying substituents (−H, −Cl, and −Br) on the aldehyde aromatic scaffold. Synthesis and characterization have been carried out, and a dimeric structure was found in the solid state. Magnetic measurements indicated that there is a ferromagnetic coupling between both Cu centers in the dimeric state and that similar conformations can be expected for the three complexes. For the model complex **C1**, the dimeric form coexists with the monomeric one in DMSO and water solutions, as clearly observed in ESI-MS and EPR, and supported by computational studies. The computational data (together with the SQUID results) also suggest that for the three complexes (**C1**, **C2**, and **C3**), the most probable dinuclear structure adopts conformation A, with a Cu–Cu distance of about ∼2.7 Å, and a dihedral angle in the metal coordination plane of 154°.

The three Cu(II) complexes have the Cu(II)/Cu(I) redox potential inside the biological redox window, and therefore they are thermodynamically able to biologically undergo a Cu(II)/Cu(I) redox cycling. Their similar ascorbate consumption rates compared to free Cu(II) confirms the potentiality of the complexes as ROS generators. The presence of the electrowithdrawing substituents on the aromatic ring (Cl or Br) shifts the Cu(II)/Cu(I) redox potential, slightly favoring the reduction from Cu(II) to Cu(I). The three complexes exhibited significant cytotoxicity in HeLa and MCF7 cancer cells, in the range of cisplatin and improved values with respect to carboplatin. The most interesting feature relies on the higher toxicity displayed by **C1** in cancer cells with respect to normal cells, most likely owing to its demonstrated high in vitro ROS production capabilities. In terms of the biological target, the studies with DNA suggest that complexes **C1**–**C3** show a moderate noncovalent binding to the double-strand DNA, but an interesting redox-dependent cleaving capacity.

The results altogether place **C1** as a promising cytotoxic agent to be further explored, whose ROS-mediated mechanism of action might produce some inherent selectivity toward cancer cells against healthy cells, giving rise to less undesired effects. Unfortunately, the nature of the halogen substituent shows no influence on the in vitro cytotoxicity of the complexes, and it also results in some solubility issues. Nonetheless, the promising in vitro outcome observed for **C1** encourage us to keep working on improving the properties of this metallic core to position it as a promising anticancer candidate.

## Experimental Section

### Chemicals

Reagents like copper(II) chloride, copper(II) acetate, DCFDA, calf thymus DNA (ct-DNA) sodium salt, benzene-1,2-diamine, *N*-chlorosuccinimide (NCS), 2-hydroxybenzaldehyde, *p*-toluenesulfonic acid (*p*-TsOH), bromine, and 2-amino-2-(hydroxymethyl)propane-1,3-diol (TRIS) were obtained from Sigma-Aldrich and Thermo Fisher. Solvents such as acetonitrile (ACN), methanol (MeOH), ethanol (EtOH), ether, chloroform (CHCl_3_), dimethyl sulfoxide (DMSO), ethyl acetate (EtOAc), dichloromethane (DCM), acetic anhydride, and hexane were used at synthesis grade purity and directly from commercial sources (Scharlab, Panreac and VWR).

### Synthesis of Ligand Precursors

#### *N*-(2-Aminophenyl)acetamide (**1**)^59^

Acetic anhydride (5.12 mL, 51.2 mmol) was added dropwise at 0 °C under a N_2_ atmosphere to a solution of benzene-1,2-diamine (5.54 g, 51.2 mmol) in anhydrous DCM (75 mL). The mixture was stirred for 2 h at 0 °C and then stored at −35 °C overnight. The precipitate was filtered off and washed with cold DCM (3 × 5 mL) and ether (3 × 5 mL) to yield 1.01 g of a white solid. The filtrate was further concentrated to the half of its volume and stored at −35 °C for 48 h more. The new precipitate was filtered off to render additional 1.56 g of the product. Yield: 38% (2.57 g). ^1^H NMR (360 MHz, DMSO-*d*_6_): δ 9.12 (s, 1H), 7.14 (d, *J* = 7.8 Hz, 1H), 6.88 (t, *J* = 7.4 Hz, 1H), 6.70 (d, *J* = 8.0 Hz, 1H), 6.52 (t, *J* = 7.3 Hz, 1H), 4.85 (s, 2H), 2.03 (s, 3H).

#### 5-Chloro-2-hydroxybenzaldehyde (**2**)^60^

Water (4 mL) was added to 2-hydroxybenzaldehyde (488 mg, 4.0 mmol). Under magnetic stirring, NCS (536 mg, 4.01 mmol, 1 equiv), *p*-TsOH (764 mg, 4.0 mmol), and NaCl (355 mg, 6.1 mmol, 1.5 equiv) were added at room temperature. The final solution was stirred at 40 °C for 1 h. Water (3 mL) was added and the formed precipitate was filtered off and washed with water (2 × 2 mL). Then, the solid was extracted with DCM and dried with sodium sulphate to afford an off-white solid. Titled compound was obtained after column chromatography (hexane/EtOAc 6:1). Yield: 14% (85 mg). ^1^H NMR (360 MHz, CDCl_3_): δ 10.94 (s, 1H), 9.87 (s, 1H), 7.56 (s, 1H), 7.49 (d, *J* = 8.4 Hz, 1H), 6.99 (d, *J* = 8.2 Hz, 1H).

#### 5-Bromo-2-hydroxybenzaldehyde (**3**)^61^

To a solution of 2-hydroxybenzaldehyde (0.5 g, 4.0 mmol) in chloroform (10 mL), bromine (0.65 g, 4.0 mmol) in chloroform (5 mL) was added dropwise over a period of 15 min at 0 °C. The resulting mixture was stirred overnight at 50 °C. Then, the reaction was diluted in water (20 mL) and extracted with chloroform (3 × 8 mL). The organic phases were combined, extracted with water (8 mL) and brine (8 mL), dried over Na_2_SO_4_, and the solvent removed under reduced pressure. The crude solid was powdered and washed with hexane (2 × 2 mL) and ether (2 × 3 mL) and the solvents were decanted. **3** was obtained without further purification. Yield: 55% (430 mg). ^1^H NMR (250 MHz, DMSO-*d*_6_): δ 10.95 (s, 1H), 10.22 (s, 1H), 7.72 (d, *J* = 2.6 Hz, 1H), 7.65 (dd, *J* = 8.8, 2.6 Hz, 1H), and 6.99 (d, *J* = 8.8 Hz, 1H).

### Synthesis of Ligands **H**_**2**_**L1**–**H**_**2**_**L3**

#### (*E*)-*N*-(2-(2-Hydroxybenzylideneamino)phenyl)acetamide (**H**_**2**_**L1**)

2-Hydroxybenzaldehyde (43.5 mg, 0.36 mmol) in absolute EtOH (6 mL) was added dropwise to a solution of **1** (58.4 mg, 0.39 mmol, 1.1 equiv) in absolute EtOH (22 mL) at 0 °C and under strong agitation. The final mixture was stirred for 15 min at 0 °C and then overnight (12 h) at room temperature. The solution was filtered and the solvent of the filtrate removed under vacuum to afford a yellowish crude. Pure **H**_**2**_**L1** was obtained by silica gel column chromatography using a gradient elution (from DCM/hexane 1:1 to EtOAc/hexane 1:1). Yield: 39% (36 mg). *R*_*f*_ (EtOAc/hexane, 2:1) = 0.7. HR-MS (ESI^+^, MeOH): for [**H**_**2**_**L1**+H]^+^, 255.1104 (theoretical, 255.1128). ^1^H NMR (360 MHz, DMSO-*d*_6_): δ 12.76 (s, 1H), 9.54 (s, 1H), 8.87 (s, 1H), 7.77–7.57 (m, 2H), 7.49–7.33 (m, 2H), 7.27 (s, 2H), 6.97 (d, *J* = 7.7 Hz, 2H), 2.05 (s, 3H). ^13^C NMR (400 MHz, DMSO-*d*_6_): δ 168.8, 163.7, 160.7, 142.3, 133.8, 132.9, 132.6, 127.3, 126.2, 125.4, 120.2, 119.6, 119.4, 117.1, 23.8. FTIR–ATR (wavenumber, cm^–1^): 3294.31, 3055.63, 1662.29, 1613.70, 1589.52, 1573.64, 1515.92, 1443.20, 1365.24, 1304.59, 1278.20, 1225.08, 1180.92, 1150.51, 1108.70, 1033.13, 1005.30, 965.08, 939.91, 909.25, 854.86, 829.27, 779.77, 752.58, 723.25, 674.22, 642.06.

#### (*E*)-*N*-(2-(5-Chloro-2-hydroxybenzylideneamino)phenyl)acetamide (**H**_**2**_**L2**)

**2** (20 mg, 0.13 mmol) in absolute EtOH (4 mL) was added dropwise to a solution of **1** (20 mg, 0.13 mmol) in absolute EtOH (10 mL) at 0 °C and under strong agitation. The final mixture was stirred for 15 min at 0 °C and at room temperature overnight. The solution was filtered, and the solvent of the filtrate was removed to afford a yellowish crude. Pure **H**_**2**_**L2** was obtained by silica gel column chromatography using a gradient elution (from DCM/hexane 3:4 to hexane/DCM:EtOAc, 1:1:0.5). Yield: 43% (16 mg). *R*_*f*_ (EtOAc/hexane, 2:1) = 0.7. HR-MS (ESI^+^, MeOH): for [**H**_**2**_**L2** + H]^+^, 289.0708 (theoretical, 289.0738). ^1^H NMR (360 MHz, DMSO-*d*_6_): δ 12.66 (br s, 1H), 9.55 (s, 1H), 8.86 (s, 1H), 7.83 (s, 1H), 7.68 (d, *J* = 7.4 Hz, 1H), 7.45 (d, *J* = 8.7 Hz, 1H), 7.35 (d, *J* = 7.2 Hz, 1H), 7.28 (m, 2H), 7.01 (d, *J* = 8.8 Hz, 1H), 2.05 (s, 3H). ^13^C NMR (400 MHz, DMSO-*d*_6_): δ 168.8, 161.9, 159.3, 142.2, 133.2, 132.7, 131.3, 127.7, 126.1, 125.2, 123.0, 121.8, 119.2, 119.1, 23.9. FTIR–ATR (wavenumber, cm^–1^): 3274.78, 2361.39, 1662.26, 1515.30, 1593.63, 1529.51, 1479.28, 1452.05, 1358.29, 1303.45, 1280.16, 1220.51, 1176.62, 1109.38, 1090.29, 1048.24, 1011.05, 959.80, 922.81, 870.39, 819.90, 760.15, 739.44, 697.78, 654.92, 641.38.

#### (*E*)-*N*-(2-(5-Bromo-2-hydroxybenzylideneamino)phenyl)acetamide (**H**_**2**_**L3**)

Compound **3** (150 mg, 0.75 mmol, 1 equiv) in absolute EtOH (5 mL) was added dropwise to a solution of **1** (123 mg, 0.76 mmol, 1 equiv) in absolute EtOH (10 mL) at 0 °C and under stirring. The final mixture was kept under the same conditions for 15 min at 0 °C and at room temperature for additional 24 h. The solution was filtered, the precipitate was washed with DCM (2 × 3 mL), and the solvent of the filtrate was removed to afford the crude **H**_**2**_**L3**. Titled compound was obtained after purification by flash silica gel column chromatography (DCM/EtOAc, 1:1). Yield: 52% (125 mg). *R*_*f*_ (EtOAc/hexane, 2:1) = 0.7. HR-MS (ESI^+^, MeOH): for [**H**_**2**_**L3** + H]^+^, 333.0193 (theoretical, 333.0233). ^1^H NMR (250 MHz, DMSO-*d*_6_): δ 12.67 (s, 1H), 9.53 (s, 1H), 8.85 (s, 1H), 7.95 (d, *J* = 2.5 Hz, 1H), 7.67 (d, *J* = 6.9 Hz, 1H), 7.56 (dd, *J* = 8.8, 2.5 Hz, 1H), 7.40–7.22 (m, 3H), 6.96 (d, *J* = 8.8 Hz, 1H), 2.07 (s). ^13^C NMR (400 MHz, DMSO-*d*_6_): δ 171.0, 164.1, 161.9, 144.2, 138.2, 136.5, 134.9, 129.9, 128.4, 127.4, 124.5, 121.7, 121.4, 112.6, 26.1. FTIR–ATR (wavenumber, cm^–1^): 3295.33, 1661.38, 1614.48, 1587.78, 1566.65, 1528.15, 1472.56, 1451.73, 1368.30, 1355.88, 1307.60, 1277.36, 1219.00, 1175.25, 1130.46, 1111.42, 1076.79, 1038.90, 1016.55, 960.34, 937.67, 914.61.

### Synthesis of Cu(II) Complexes

#### Complex **C1** ([Cu(**L1**)]_2_)

Cu(OAc)_2_·2H_2_O (15.7 mg, 0.08 mmol, 1 equiv) in ACN (3 mL) was slowly added to a solution of **H**_**2**_**L1** (20 mg, 0.08 mmol, 1 equiv) in ACN (8 mL) at room temperature. The final mixture was stirred for 2 h and the formed precipitate was filtered off and washed with ACN (2 × 3 mL) and with Et_2_O (2 × 3 mL). The solid so obtained was identified as **C1**. Yield: 68% (17 mg). HR-MS (ESI^+^, DMSO–MeOH): for [**C1** + H]^+^, 631.0456 (theoretical, 631.0462); for [**C1** + Na]^+^, 653.0194 (theoretical, 653.0282). Elemental Analysis Calcd for **C1** (C_30_H_24_Cu_2_N_4_O_4_): C, 57.05; H, 3.83; N, 8.87. Found: C, 56.61; H, 3.81; N, 8.56. FTIR–ATR (wavenumber, cm^–1^): 2363.09, 1610.64, 1477.82, 1458.40, 1429.20, 1401.49, 1376.22, 1353.19, 1326.39, 1281.82, 1244.19, 1217.10, 1173.53, 1145.83, 1126.54, 1026.08, 961.53, 922.74, 849.42, 793.31, 747.55, 679.15, 649.79, 620.37.

#### Complex **C2** ([Cu(**L2**)]_2_)

Cu(OAc)_2_·2H_2_O (6.0 mg, 0.03 mmol, 1 equiv) in ACN (2 mL) was slowly added to a solution of **H**_**2**_**L2** (9 mg, 0.03 mmol, 1 equiv) in ACN (5 mL) at room temperature. The same procedure as for **C1** was followed to obtain pure **C2**. Yield: 73% (8 mg). HR-MS (ESI^+^, DMSO–MeOH): for [**C2** + H]^+^, 700.9661 (theoretical, 700.9839). Elemental Analysis Calcd for **C2** (C_30_H_22_Cl_2_Cu_2_N_4_O_4_): C, 51.44; H, 3.17; N, 8.00. Found: C, 51.47; H, 3.18; N, 7.66. FTIR–ATR (wavenumber, cm^–1^): 1614.62, 1492.27, 1475.85, 1406.65, 1375.34, 1318.32, 1281.53, 1240.53, 1201.84, 1159.66, 1129.77, 1027.64, 988.20, 960.72, 932.29.

#### Complex **C3** ([Cu(**L3**)]_2_)

Cu(OAc)_2_·2H_2_O (24.0 mg, 0.12 mmol, 1 equiv) in ACN (3 mL) was slowly added to a solution of **H**_**2**_**L3** (40 mg, 0.12 mmol, 1 equiv) in ACN/DCM (1:1, 12 mL) at room temperature. The same procedure as for **C1** was followed to obtain pure **C3**. Yield: 74% (35 mg). HR-MS (ESI^+^, DMSO–MeOH): for [**C3** + H]^+^, 786.8678 (theoretical, 786.8673). Elemental Analysis Calcd for **C3** (C_30_H_22_Br_2_Cu_2_N_4_O_4_): C, 45.64; H, 2.81; N, 7.10. Found: C, 45.41; H, 2.81; N, 6.82. FTIR–ATR (wavenumber, cm^–1^): 1613.76, 1492.89, 1475.85, 1454.37, 1436.29, 1408.34, 1374.91, 1317.00, 1281.65, 1241.56, 1203.98, 1159.97, 1133.05, 1070.82, 1028.23, 988.61, 961.31.

### Physical Measurements. Instruments and Experimental Procedures

#### SQUID Data

Magnetic characterization has been performed using a conventional SQUID magnetometer MPMS-XL from Quantum Design working at a magnetic field up to 5 T and temperature down to 2 K. The samples (powder) are filled in polypropylene sleeves then sealed in order to remove the maximum of dioxygen, which give the signal around 50 K (antiferromagnetic transition). However, despite such care, the oxygen signal is visible in the **C1** sample, but **C2** and **C3** are rather clean. Diamagnetic contribution of the sample holder was removed. The susceptibility was fitted using the Bleaney–Bowers formula of two coupled *S* = 1/2.^31^

The isothermal (*T* = 2 K) magnetization was fitted using the Brillouin function with one *S* = 1 (equivalent to two coupled *S* = 1/2 at *T* < 2*J*) and two uncoupled *S* = 1/2

#### NMR Spectrometry

NMR experiments were recorded on BRUKER DPX-250, 360, and 400 MHz instruments at the Servei de Ressonància Magnètica Nuclear (UAB). Deuterated solvents were directly purchased from commercial suppliers. All spectra have been recorded at 298 K. The abbreviations used to describe signal multiplicities are: s (singlet), bs (broad singlet), d (doublet), dd (double doublet), and m (multiplet). All ^13^C NMR acquired spectra are proton decoupled.

#### ESI-MS Measurements

HR ESI-MS measurements were recorded after diluting the corresponding solid complexes using a MicroTOF-Q (Brucker Daltonics GmbH, Bremen, Germany) instrument equipped with an electrospray ionization source (ESI) in positive mode at the Servei d’Anàlisi Química (UAB). The nebulizer pressure was 1.5 bar, the desolvation temperature was 180 °C, flow rate of dry gas was 6 L min^–1^, the capillary counter electrode voltage was 5 kV, and the quadrupole ion energy was 5.0 eV.

#### EPR Experiments

EPR measurements were carried out on a BRUKER ELEXSYS 500 X-band CW-ESR spectrometer, with an ELEXSYS Bruker instrument equipped with a BVT 3000 digital temperature controller. The spectra were recorded at 120 K in frozen DMSO solutions otherwise noticed. Typical parameters were: a microwave power of 10–20 mW, a modulation frequency of 100 kHz, and a modulation gain of 3 G. EPR spectra were simulated using the EasySpin toolbox developed for Matlab.^62^ Copper spin quantification has been carried out for **C1** in frozen DMSO solutions (0.5 mM, e.g., 1 mM copper concentration, with or without 0.1 M [NBu_4_][PF_6_] (TBAP) electrolyte) through double integration of the EPR derivative signal, using standardized Cu(NO_3_)_2_ solutions as an external calibrator.

#### Cyclic Voltammetry

Cyclic voltammograms were taken on a BioLogic SP-150 potentiostat and using EC-Lab 5,40 software. DMSO was used as a solvent with 0.1 M of [NBu_4_][PF_6_] (TBAP) as a supporting electrolyte. Measurements were carried out with a three-electrode configuration cell: glassy carbon electrode as the working electrode, Ag wire in a 0.1 M TBAP solution in DMSO (semielectrode) as the reference electrode, and Pt as the counter electrode. The ferrocene (Fc^+^/Fc) system was used as the internal standard. The scan rate (ν) varied between 300 and 25 mV·s^–1^. All the experiments were recorded under an argon atmosphere.

#### Elemental Analysis

C, H, and O analyses were performed at the Servei d’Anàlisi Química (UAB) on a Flash EA 2000 CHNS Thermo Fisher Scientific equipment, with a TCD and a MAS 200 R autosampler for solid samples.

#### IR Spectroscopy

Attenuated total reflectance (ATR)–FTIR spectra were recorded on a PerkinElmer spectrometer, equipped with a universal ATR accessory, with a diamond window in the range 4000–650 cm^–1^.

#### UV–Vis Characterization

All the spectra were recorded at room temperature either on an Agilent HP 8453, Varian Cary 50 Bio, a Varian Cary 60 Bio, or a PerkinElmer Lambda 650 spectrophotometer, using 1 cm quart-cuvettes. Noncovalent DNA–complex interactions were studied by UV–vis measurements. Solutions of complexes **C1**–**C3** were prepared in 50 mM NaCl/5 mM Tris–HCl buffer (pH 7.2), containing a maximum of 5% DMSO to solubilize them. ct-DNA stock solutions were prepared from their corresponding sodium salt and the concentration was determined from its absorbance at 260 nm (ε = 6600 cm^–1^). Blank and dilution effects were corrected. Ascorbate consumption experiments were monitored by UV–vis at the maximum absorption band of the ascorbic acid (100 μM) at 265 nm for about 45 min. CuCl_2_ and the assayed complexes **C1**–**C3** were added at a final concentration of 2 μM in 50 mM NaCl/5 mM Tris–HCl buffer (pH 7.2), with a maximum of 5% of DMSO.

#### Circular Dichroism

CD experiments were acquired on a JASCO 715 spectropolarimeter. Measurements were carried out at a constant temperature of 20 °C. CD spectra were measured in 50 mM NaCl/5 mM Tris–HCl buffer (pH 7.2). The ct-DNA concentration was 50 μM. Different samples with increasing amounts of the complexes to study (0, 50, 100 μM) were incubated at 37 °C for 24 h, containing a maximum of 5% DMSO to solubilize them. Ct-DNA stock solutions were prepared from their corresponding sodium salt (Sigma-Aldrich) and the concentration was determined from their absorbance at 260 nm (ε = 6600 cm^–1^).

#### DNA-Cleaving Experiments

Gel electrophoresis experiments were performed on agarose gel (1% in Tris–acetate EDTA (TAE) buffer), using a BIORAD horizontal tank connected to a variable potential power supply. Samples were stained with EB and revealed with a Super GelDoc PlusImager. Complexes **C1**–**C3** were incubated with the plasmid DNA (200 ng of BlueScript plasmid per well) in 20 mM NaCl/40 mM Tris–HCl buffer (pH 7.20) medium for 24 h at 37 °C (<10% DMSO in the final mixture to solubilize the complexes). Samples containing the reducing agent ascorbic acid were incubated for 1.5 extra hours in the presence of ascorbic acid (100 μM).

#### Cell Viability Assays

The IC_50_ values were evaluated using the PrestoBlue Cell Reagent (Life Technologies) assay. Working concentrations of complexes **C1**–**C3** (final amount <0.1% DMSO in biological experiments) were prepared in the corresponding MEM (modified Eagle’s medium, Invitrogen) for each cell. Human cancer cells (HeLa and MCF7) and nontumoral NIH 3T3 cells were obtained from American Type Culture Collection (ATCC, Manassas, VA, USA). HeLa cells were routinely cultured with MEM; MCF7, with DMEN-F12 (Dulbecco’s MEM/Nutrient Mixture F-12 Ham); and NIH 3T3, with DMEM (Dulbecco’s MEM), all containing 10% heat-inactivated fetal bovine serum at 37 °C in a humidified CO_2_ atmosphere. Cells were plated at a density of 3 × 10^3^ cells/well in 100 μL of culture medium and allowed to grow overnight. After the required incubation time with different concentrations (0, 1, 5, 10, 25, 50, 100, or 200 μM) of each complex, 10 μL of PrestoBlue were added following the standard protocol. The fluorescence of each well was measured at 572 nm with a Microplate Reader Victor3 (PerkinElmer). The relative cell viability (%) for each sample related to the control well was calculated. Each complex was tested per triplicate and averaged from three independent sets of experiments. Blank and complex controls were also considered.

#### Intracellular ROS Production Assays

HeLa cells were plated and allowed to adhere overnight in a 96-well plate (2 × 10^4^ cells/well). The DCFDA reagent (25 μM in DMSO) was then added and the cells incubated at 37 °C in the dark for 30 min. The DCFDA solution was removed and cells were treated with the compounds at the corresponding IC_50_ values (at 72 h) and incubated for 4 h. The experiments were run in triplicate. H_2_O_2_ was used as a positive control at 100 μM. The fluorescence of each well was measured at 535 nm with a Microplate Reader Victor3 (PerkinElmer) after excitation at 485 nm.

#### In Vitro Apoptosis Assays

Induction of apoptosis was determined by a flow cytometric assay with Annexin V–fluorescein isothiocyanate (FITC) by using an Annexin V–FITC apoptosis detection kit (Roche). Exponentially growing HeLa cells in 6-well plates (3 × 10^5^ cells/well) were exposed to concentrations equal to the IC_50_ for 24 h (70 μM), determined prior to the experiment. After the cells had been stained with the Annexin V–FITC and propidium iodide, the percentage of apoptotic cells was analyzed by flow cytometry (FACS Calibur).

### Computational Details

The geometry of the monomeric [Cu^II^(**L1**)(DMSO)] and dimeric [Cu^II^(**L1**)]_2_ complexes was optimized with Gaussian 09^63^ at the DFT theory level using the hybrid B3LYP functional combined with Grimme’s D3 correction^64^ for dispersion and the split-valence plus polarization function 6-31g(d,p) basis-set for the main group elements, SDD plus *f*-functions^65^ and pseudopotential were applied for copper. The effect of solvation was taken into account using the SMD continuum model of Marenich et al.^66^ For all the structures, minima were verified through frequency calculations.

The thermodynamic stability in solution was estimated computing the Gibbs free energy change using the implicit solvent continuum model.^67^ Concerning the 1e^–^ reduction products, the previously optimized geometry of the Cu(II) complexes [Cu^II^(**L1**)(DMSO)] was reoptimized at the same level of theory imposing the multiplicity relative to the [Cu^I^(**L1**)(DMSO)]^−^ and [Cu^II^(**L1**^•–^)(DMSO)]^−^ forms. The Gibbs free energy values were obtained by the addition of the thermal and entropic corrections (*G*^therm^), obtained in the optimization stage, to the potential energy value of single point calculations with the extended basis-set def2-TZVP for the main group elements^67^ and the quadruple-ζ def2-QZVP basis set for Cu.^68,69^

The *g* and *A* tensors of the ^63^Cu center for each complex were obtained using the method implemented into the Orca package.^70,71^ The *A* tensor is obtained as a sum of the three contributions: the isotropic Fermi contact (*A*^FC^), the anisotropic dipolar (*A*_*x*,*y*,*z*_^D^), and the spin–orbit coupling term (*A*_*x*,*y*,*z*_^SO^). *A* tensors were computed using the functional B3LYP, while for *g* PBE0 was used, both coupled with a triple-ζ basis set 6-311g(d,p).^35^

The exchange coupling constants *J* for the dinuclear **C1** complex were calculated with the functional B3LYP and the 6-311g basis set with the software ORCA,^70^ according to the method reported in the literature.^72^ Using *S*_1_ = *S*_1_ = 1/2 in the Heisenberg Hamiltonian *Ĥ* = *Ŝ*_1_·*Ŝ*_2_, the value of *J* can be expressed as: *J* = *E*_BS_ – *E*_HS_, where *E*_BS_ and *E*_HS_ are the energies of the broken-symmetry solution and the triplet state.

UV–vis vertical excitations were simulated on the time-dependent DFT framework using the solvent continuum model.^73^ The simulations were carried out on the previously optimized geometries in solvent using BH and HLYP functionals and the triple-ζ type def2-TZVP basis set, according to the method established previously.^74^ The predicted electronic spectrum of **C1** was generated using Gabedit software.^75^

## Supporting Information Available

NMR, ESI-MS, FT-IR, EPR, UV–vis absorbance, and SQUID data of ligands and complexes; cell viability results; EPR parameters obtained; and Gibbs energy values computationally calculated and flow cytometry of HeLa cells (PDF)
