# Supplementary material for: Copper(II) N,N,O-Chelating Complexes as Potential Anticancer Agents
Source: Inorg Chem. 2021 Feb 17;60(5):2939–52. doi: 10.1021/acs.inorgchem.0c02932 (PMC8483446; doi:10.1021/acs.inorgchem.0c02932)
Supplement: Supplementary file 1 — ic0c02932_si_001.pdf [file ic0c02932_si_001.pdf]

SUPPORTING MATERIAL

Copper(II) *N*,*N*,*O*-chelating complexes as potential anticancer agents

Quim Peña,[a,b,#] Giuseppe Sciortino,[a,c] Jean-Didier Maréchal,[a] Sylvain Bertaina,[d] A. Jalila Simaan,[b] Julia Lorenzo,[e] Mercè Capdevila,[a] Pau Bayón,[a] Olga Iranzo[b,]* and Òscar Palacios[a,]*

[a] *Departament de Química, Facultat de Ciències, Universitat Autònoma de Barcelona, 08193-Cerdanyola del Vallès, Barcelona, Spain.*

[b] *Aix Marseille Univ, CNRS, Centrale Marseille, iSm2, Marseille, France.*

[c] *Institute of Chemical Research of Catalonia (ICIQ), Av. Països Catalans 16, 43007 Tarragona, Spain*

[d] *Aix Marseille Univ., CNRS, IM2NP, Marseille, France.*

[e] *Institut de Biotecnologia i Biomedicina, Dept. Bioquímica i Biologia Molecular, Universitat Autònoma de Barcelona, 08193-Cerdanyola del Vallès, Barcelona, Spain.*

*correspondence to: olga.iranzo@univ-amu.fr ; oscar.palacios@uab.cat

FOOTNOTE

[#] Current Address: *Department of Nanomedicine and Theranostics, Institute for Experimental Molecular Imaging, Faculty of Medicine, RWTH Aachen University, Aachen, Germany*

Figure S1. (**A**) ^{1}H NMR (360 MHz, d_6-DMSO), (**B**) ^{13}C NMR (400 MHz, d_6-DMSO) and (**C**) FT-IR spectra of compound **H_2L1**.

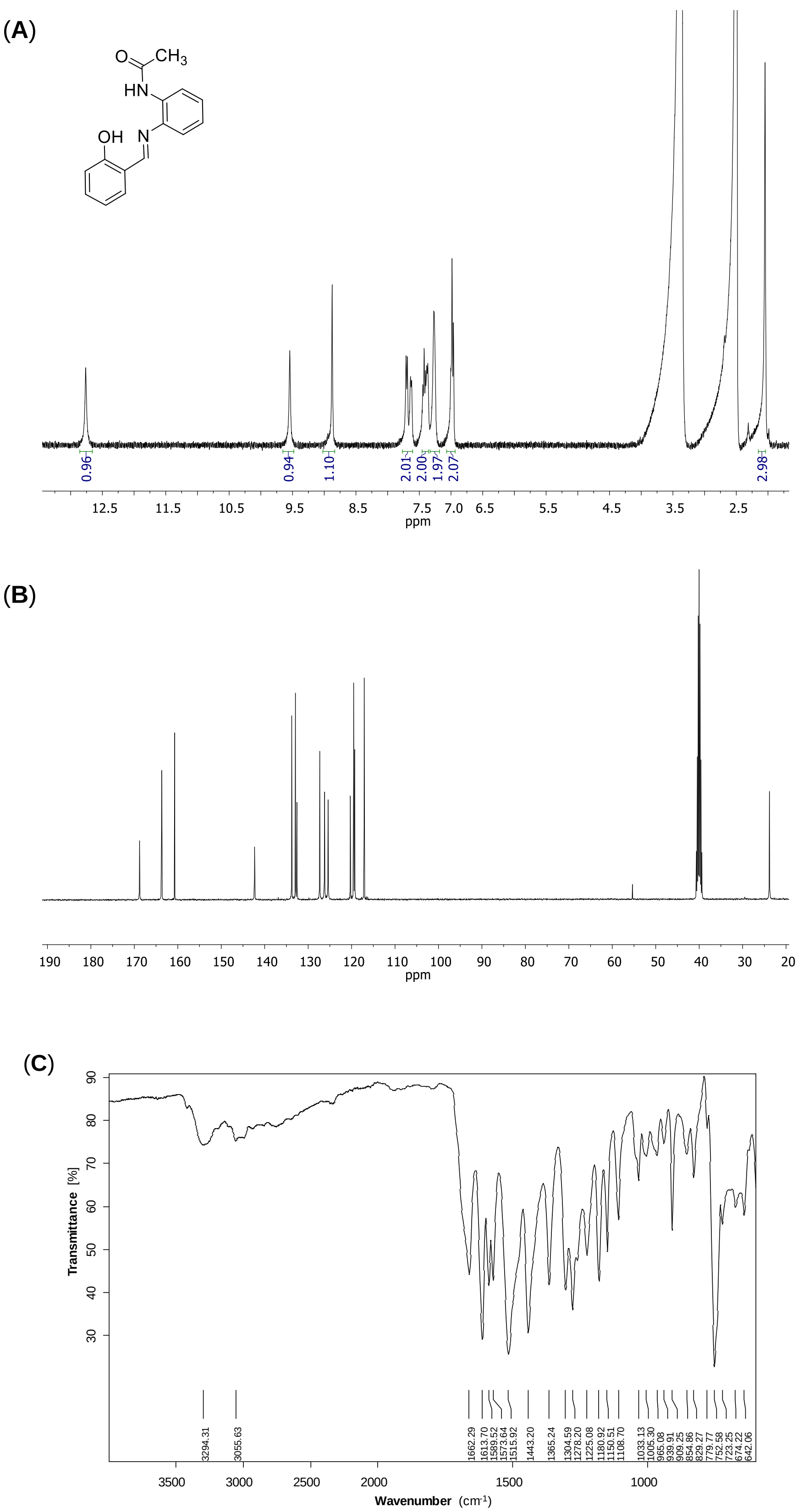

Figure S2. (**A**) ^{1}H NMR (360 MHz, d_6-DMSO), (**B**) ^{13}C NMR (400 MHz, d_6-DMSO) and (**C**) FT-IR spectra of compound **H_2L2**.

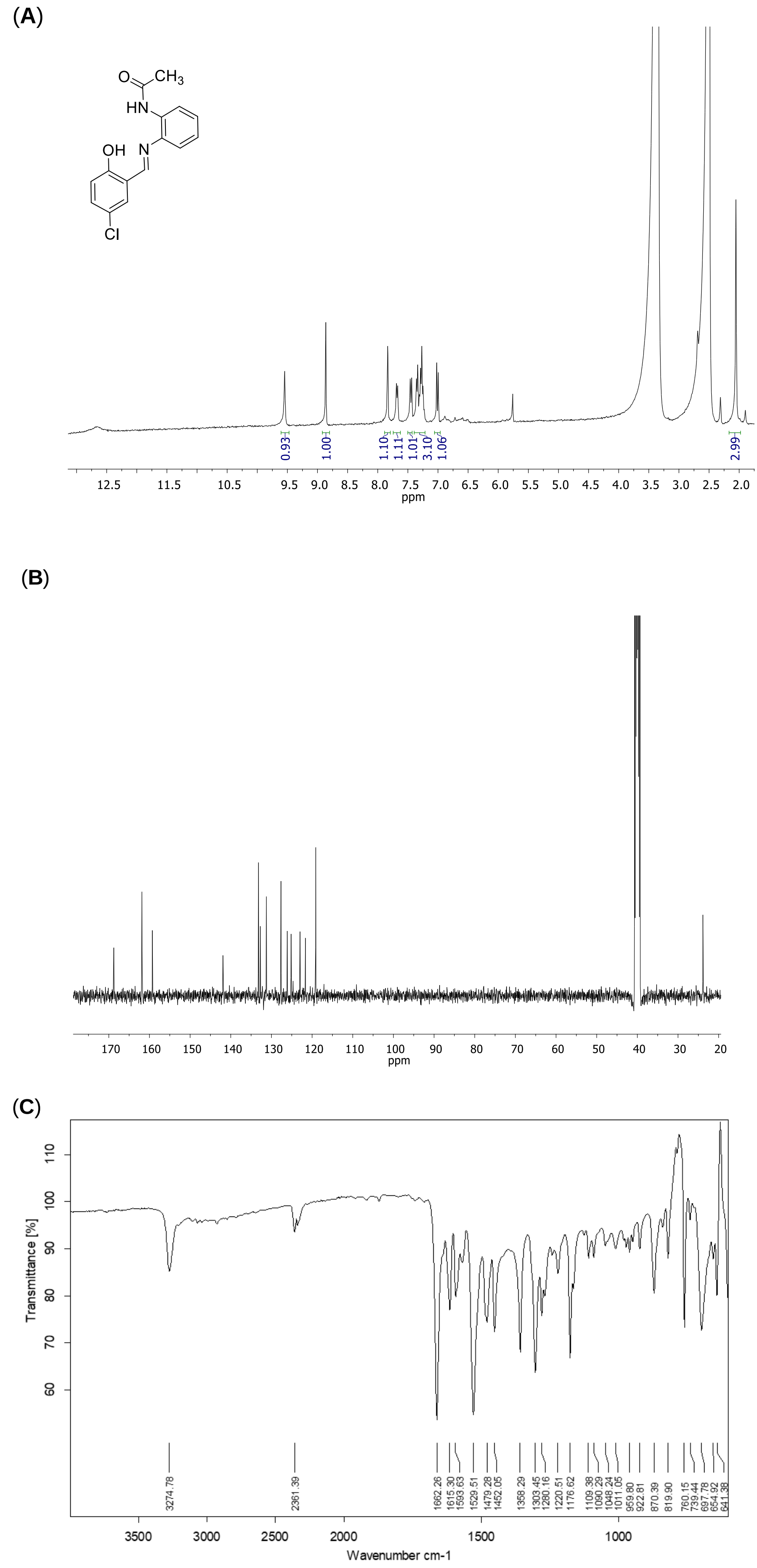

Figure S3. (**A**) ^{1}H NMR (250 MHz, d_6-DMSO), (**B**) ^{13}C NMR (400 MHz, d_6-DMSO) and (**C**) FT-IR spectra of compound **H_2L3**.

(**A**)

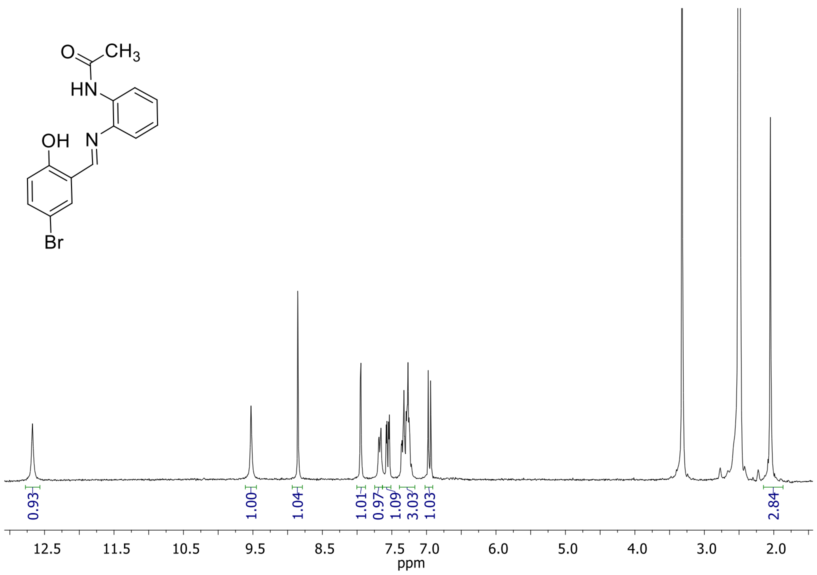

(**B**)

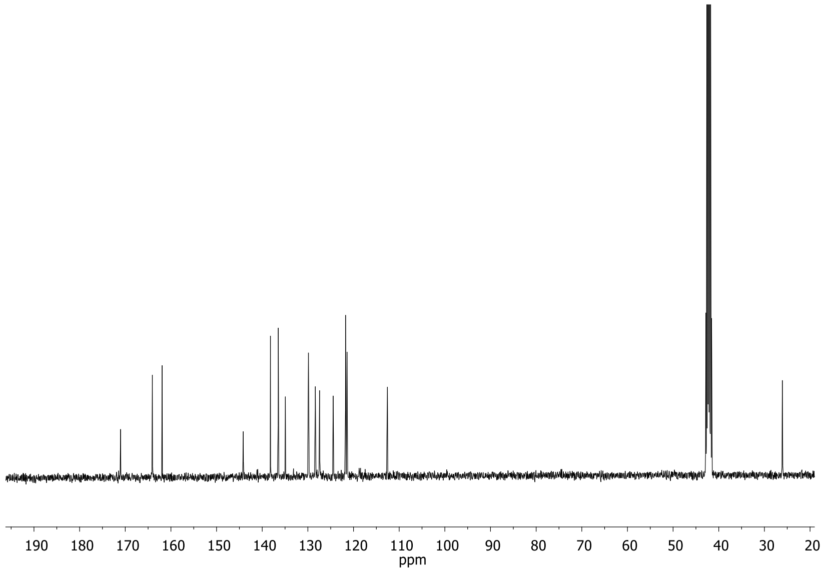

(**C**)

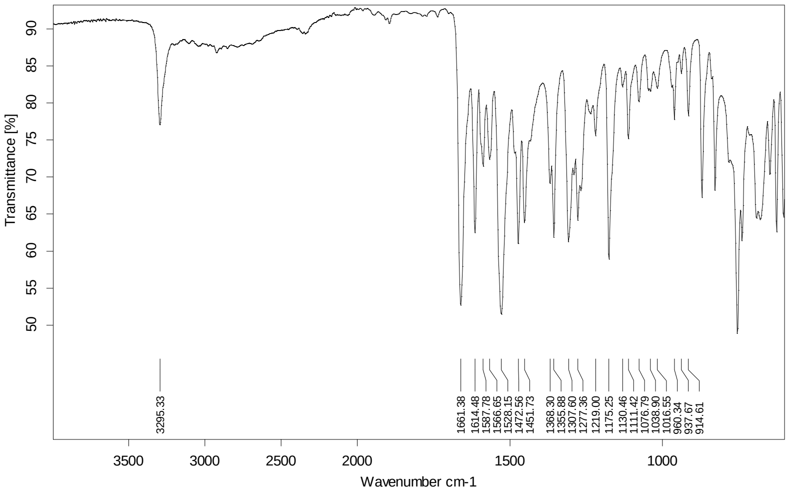

Figure S4. Characterization of the Cu(II) complexes **C1-C3** by (**A**) HR-ESI-MS (ESI^+, in MeOH-DMSO 90:10) and IR, as well as (**B**) their EPR spectra, recorded at 120 K in frozen DMSO solution. (**C**) Full ESI-MS spectrum of **C1** (ESI^+, in MeOH-DMSO 90:10) confirming the coexistence of the monomeric and the dimeric forms.

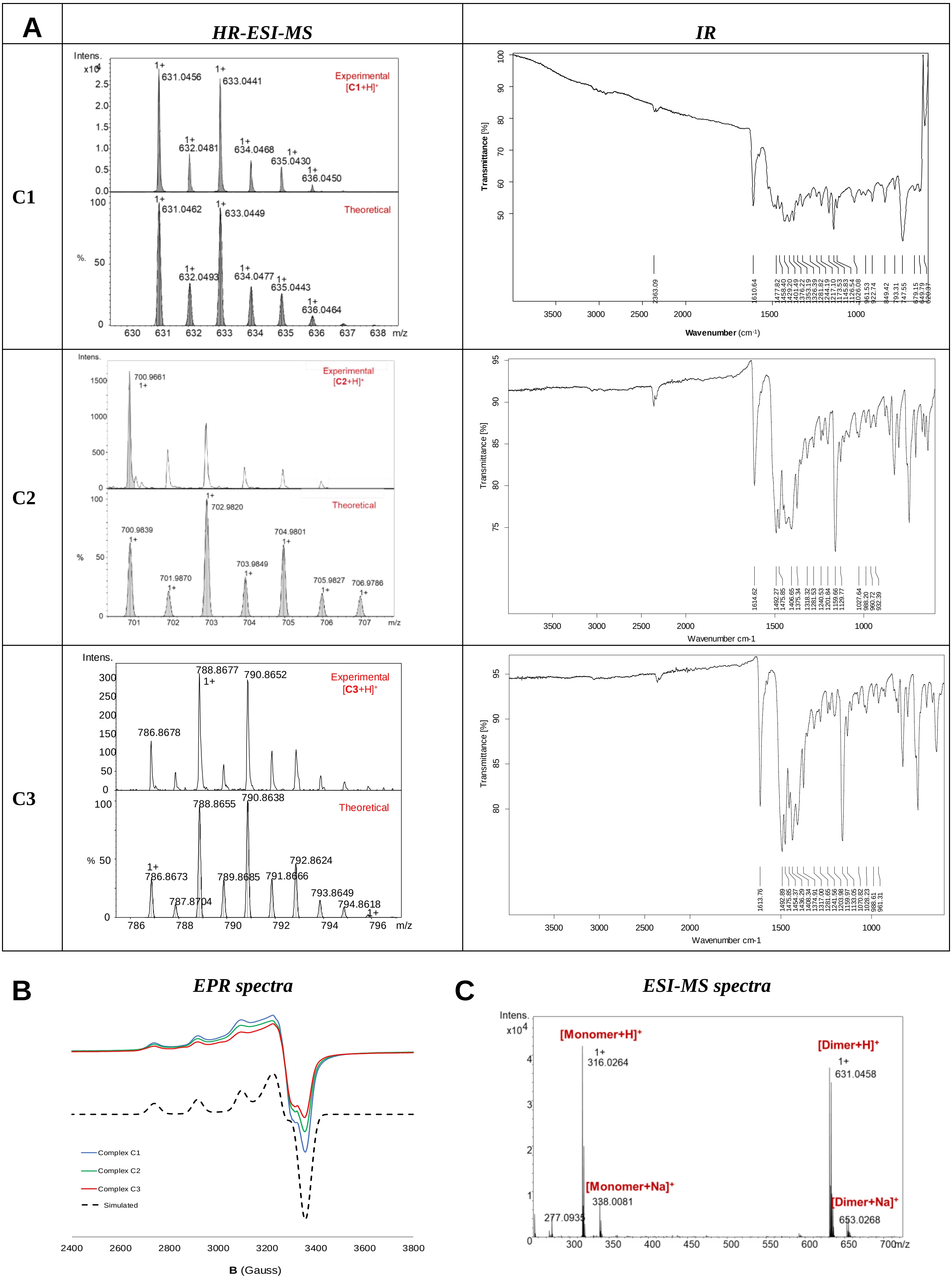

Figure S5. SQUID data obtained for complexes **C1** and **C2**: (**A**) susceptibility measured at 1T (red curve is the best fit for a dimeric complex); (**B**) AC - magnetic susceptibility; and (**C**) magnetization measured at 2K showing a fitting with the theoretical model for 2 coupled spins.

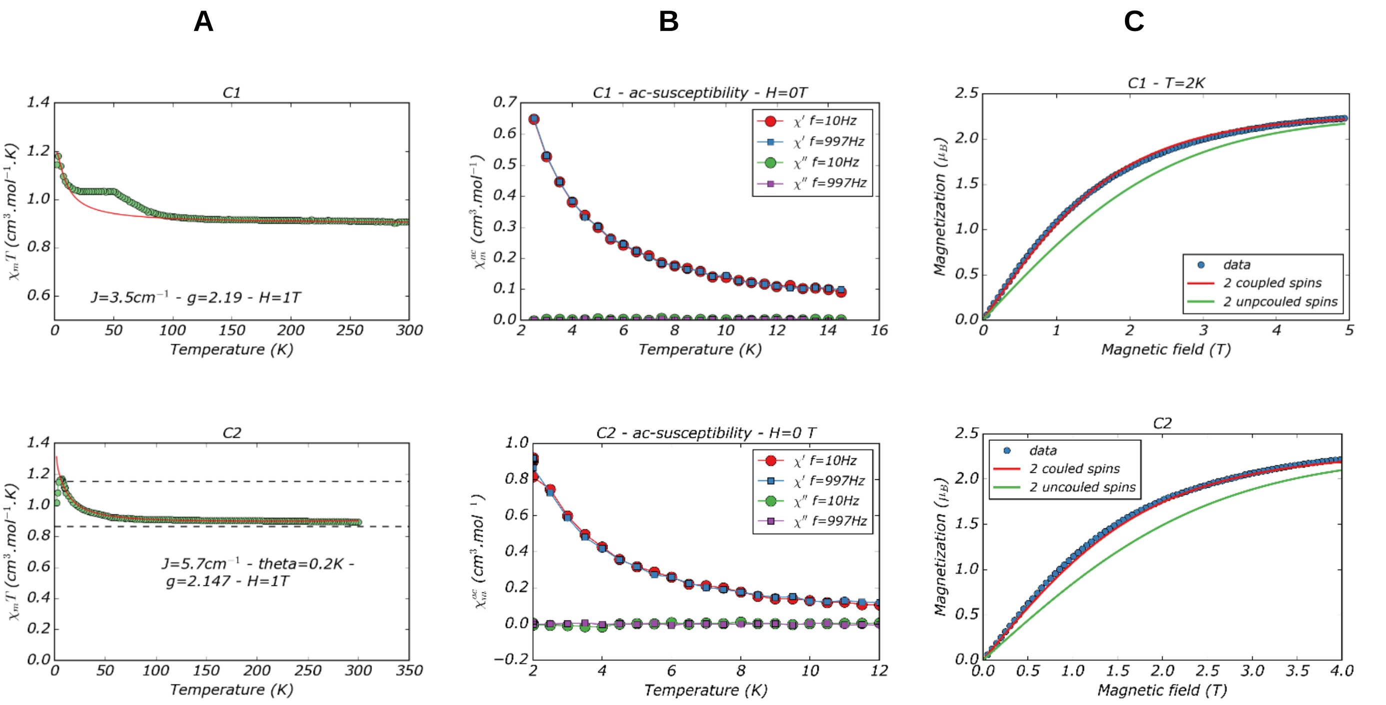

Figure S6. Comparison of the experimental absorbance spectrum of **C1 (**DMSO:water 10:90, 30 μM, dotted line) and the spectra calculated with BHandHLYP for conformations A (brown) and C (purple) of [Cu^{II}**(L1)**]$_2$ dimeric species (brown) and monomeric [Cu^{II}**(L1)**(H_2O)] (red) and [Cu^{II}**(L1)**(DMSO)] (yellow). The Y axes corresponds to normalized absorbance in order to allow better comparison.

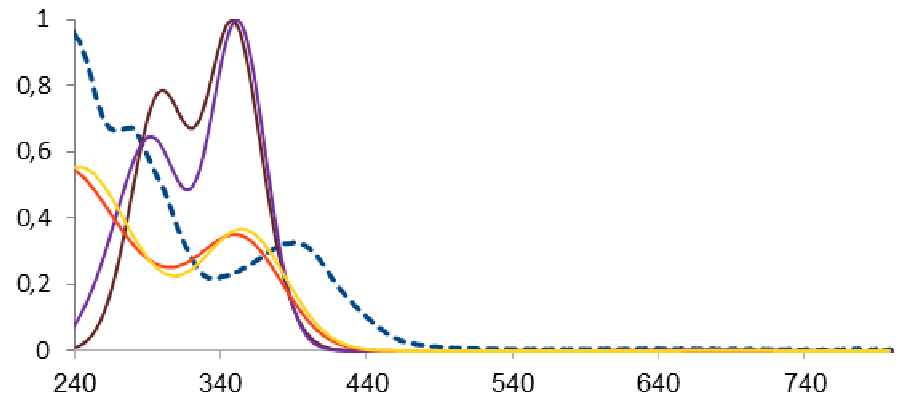

Figure S7. Comparison of the Cu(II) *d-d* bands experimental spectrum of **C1** (DMSO:water 10:90, 1.0 mM, dotted line) and the calculated spectra at BHandHLYP theory level for the conformations A (brown) and C (purple)of $[Cu^{II}(\mathbf{L1})]_2$ dimeric species (brown), $[Cu^{II}(\mathbf{L1})(H_2O)]$ (red), and $[Cu^{II}(\mathbf{L1})(DMSO)]$ (yellow) species in the range 550-800 nm.. The Y axes corresponds to normalized absorbance in order to allow better comparison.

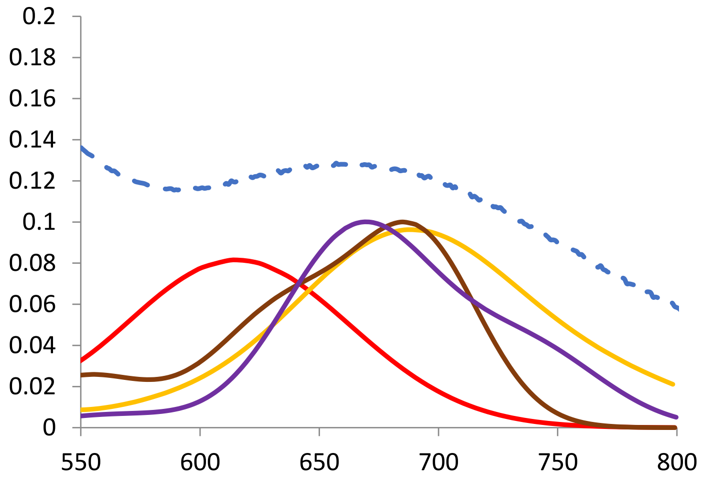

Figure S8. Linear plots of I_{pc} (blue) and I_{pa} (red) *vs.* the root square of the scan rate (ν) for complexes **C1**, **C2** and **C3** in DMSO.

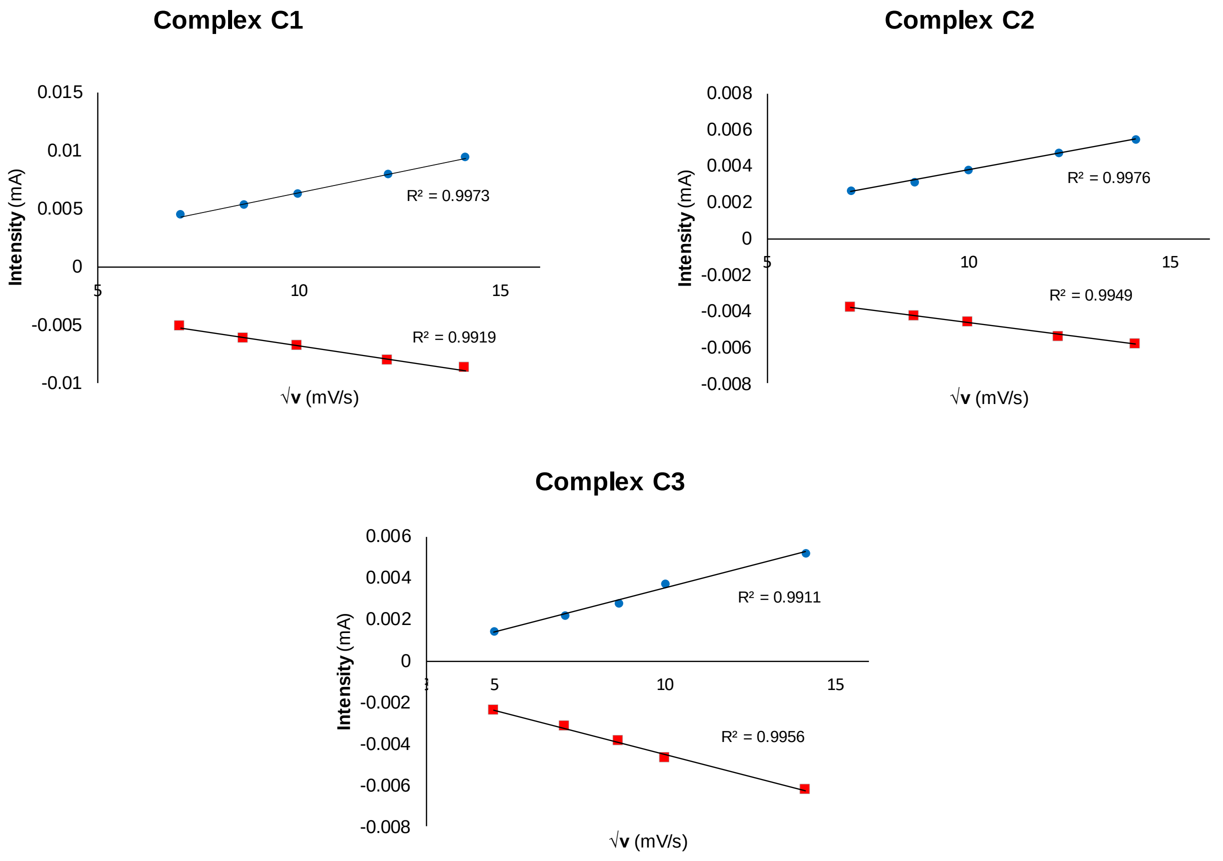

Figure S9. Optimized geometry of the Cu(II) complex species in different oxidation states: (**A**) [Cu^{II}(**L1**)(DMSO)], (**B**) [Cu^{I}(**L1**)(DMSO)]$^-$ and (**C**) [Cu^{II}(**L1**$\cdot^-$)(DMSO)]$^-$.

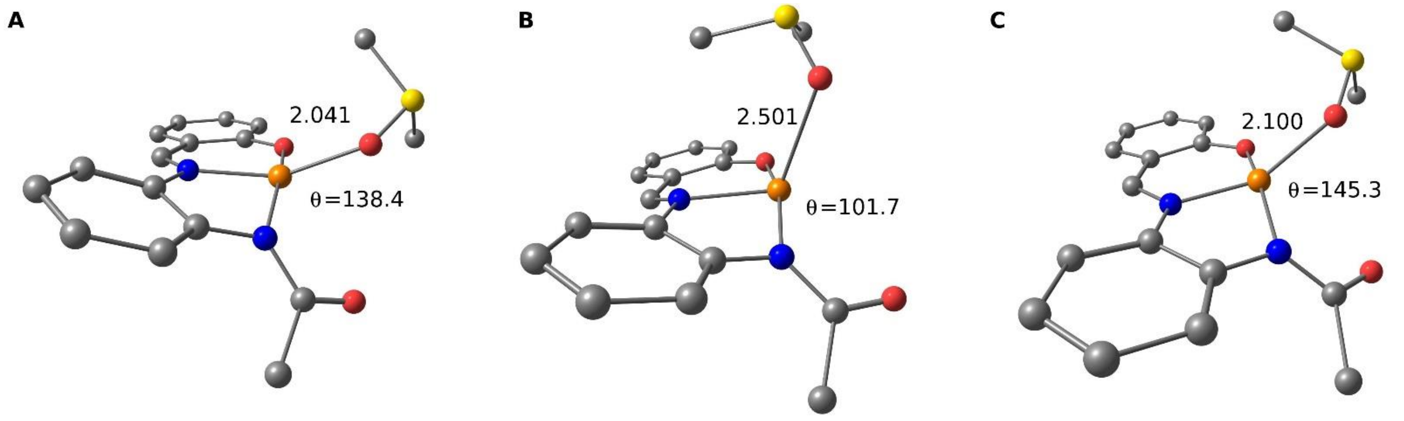

Figure S10. Cell viability assays in (**A**) HeLa cell cultures of **H_2L1**, **H_2L2** and **H_2L3**, and their corresponding Cu(II) complexes **C1**, **C2** and **C3** at different concentrations after 72 h of treatment; and (**B**) MCF7 cell cultures for **C1** and **C3** at different concentrations at 72 h. Free ligands have been also tested in MCF7 with similar profile as in HeLa (data not shown). **C2** was not able to be assessed due to solubility issues in MCF7 culture medium. The obtained values average at least three independent experiments.

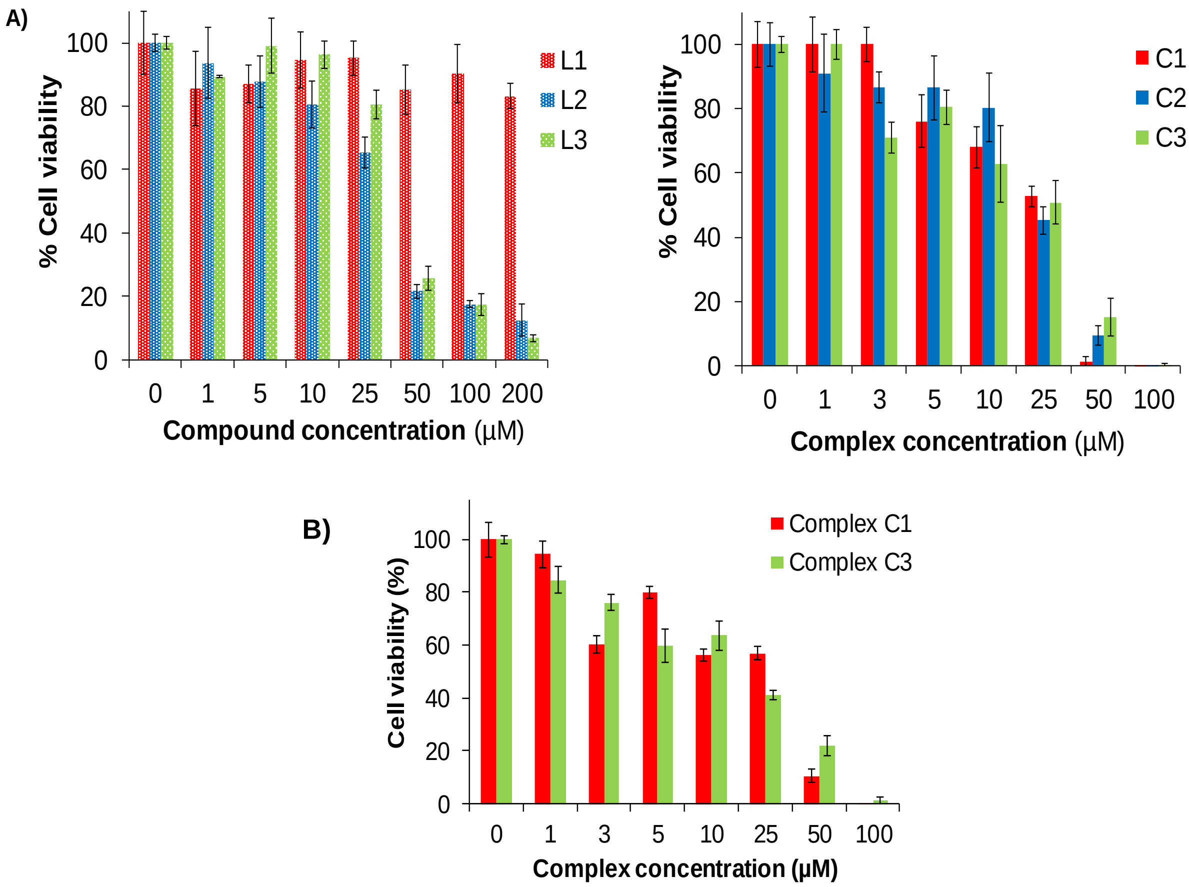

Figure S11. Plot of $A_0/(A_0-A)$ *vs.1/[DNA]* for complexes **C1**, **C2** and **C3** (the ones showing a constant change upon *ct*-DNA addition) at their absorption maxima at 397, 424 and 438 nm, respectively.

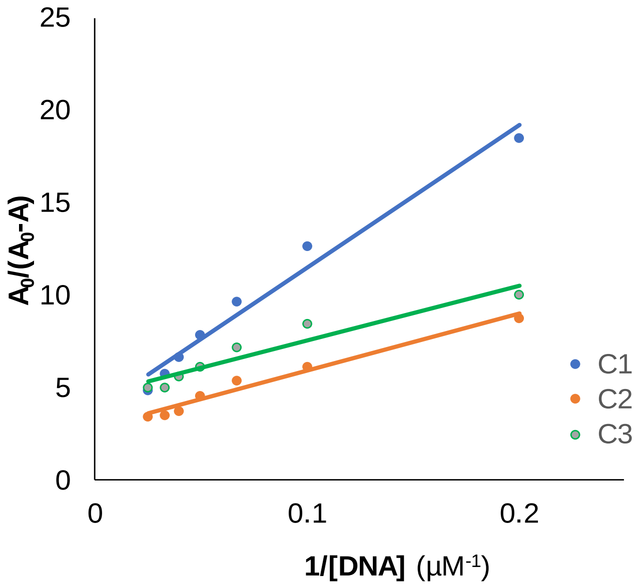

Table S1. EPR parameters for complexes **C1-C3** recorded in frozen DMSO solutions obtained from their corresponding EPR spectra, recorded at 120 K in frozen DMSO solution (Figure S4B).

complex	$g_{//}$	$A_{//}$ (cm^{-1})	$g_{\perp}$	$A_{\perp}$ (Gauss)
**C1**	2.244	$183.4 \cdot 10^{-4}$	2.043/2.076	<20-30
**C2**	2.243	$183.3 \cdot 10^{-4}$	2.043/2.076	<20-30
**C3**	2.244	$183.4 \cdot 10^{-4}$	2.043/2.076	<20-30

Table S2. Percentage of **C1** mononuclear Cu(II) species obtained from the copper spin quantification of its EPR spectra recorded at 120 K in frozen DMSO solutions with and without electrolyte (TBAP) at different time points. Spin quantification has been performed through double integration and percentages have been measured based on a Cu(II) nitrate standardized solution, used as external calibration.

Time (h)	Mononuclear Cu(II) percentage (%)	
	DMSO	DMSO containing TBAP
0	28	32
3	30	32
24	46	53
120	47	59

Table S3. Calculated and experimental transitions (λ_{max}) for the dimeric $[Cu^{II}(\mathbf{L1})]_2$ and monomeric $[Cu^{II}(\mathbf{L1})(solv)]$ species.

Species	$\lambda_{max}^{calc, a}$ (nm)	$\lambda_{max}^{exp, b}$ (nm)	absolute deviation (%)
$[Cu^{II}(\mathbf{L1})(DMSO)]$	352.7	384.99	8.4
	685.5	684.9	0.1
$[Cu^{II}(\mathbf{L1})(H_2O)]$	354.2	384.99	8.0
$[Cu^{II}(\mathbf{L1})]_2$ (Conf. A)	349.6	384.99	9.2
	685.5	684.9	0.1
$[Cu^{II}(\mathbf{L1})]_2$ (Conf. C)	352.3	384.99	8.5
	668.2	684.9	2.4

[a] Vertical transition computed at TD-DFT theory level with BHandHLYP functional combined with the basis-set def2-TZVP in smd continuum model for DMSO. *[b] Spectrum recorded in DMSO:water mixture 10:90.*

Table S4. Summary of the Cu(II)/Cu(I) redox potentials obtained for complexes **C1**, **C2** and **C3** in DMSO with 0.1 M TBAP at a scan rate of 100 mV/s *vs.* Fc^+/Fc.

**Species**	**$E_{1/2}$ (V)**	**E_{pc} (V)**	**E_{pa} (V)**	**I_a/I_c**	**ΔE_p (mV)**
Complex **C1**	-1.07	-1.15	-0.99	1.1	160
Complex **C2**	-1.03	-1.09	-0.97	0.9	120
Complex **C3**	-1.03	-1.08	-0.97	0.9	110

Table S5. Gibbs energy values for the $[Cu(\mathbf{L1})(DMSO)]^-$ product of the $1e^-$ reduction. The reduction products have been considered in S=1 (Cu^I) and S=3, corresponding to the ligand reduction ($\mathbf{L1}^{\cdot-}$). Values were computed in SMD continuum model for DMSO.

**Species**	**S**	**G (a.u.)**	**ΔG ($kcal \cdot mol^{-1}$)**
$[Cu^I(\mathbf{L1})(DMSO)]^-$	3	-3033.023686	0.0
$[Cu^{II}(\mathbf{L1}^{\cdot-})(DMSO)]^-$	1	-3032.971530	32.7

Table S6. Annexin V/PI apoptosis assay on HeLa cells by flow cytometry. We analyzed control cells (with no additives), cells treated with cisplatin at 40 µM (IC_{50}, 24 h)[213] as positive control, and cells treated with **C1** at 70 µM (IC_{50}, 24 h) for 24 h.

**Sample**	**% Live cells (Q1)**	**% Early apoptosis (Q2)**	**% Late Apoptosis/ Necrosis (Q3)**	**% Damaged cells (Q4)**
Control	99.8	-	-	-
Cisplatin	45	23	22	10
Complex **C1**	50	12	24	14
